# From passive sealing to active microenvironment remodeling: design strategies, biological effects, and translational challenges of multifunctional hydrogels for postoperative pancreatic fistula prevention

**DOI:** 10.3389/fbioe.2026.1942858

**Published:** 2026-09-25

**Authors:** Dongfang Yang, Yutong Hu, Hao Fu, Xiaoxia Sun, Zhongxing Miao

**Affiliations:** 1 The Second Affiliated Hospital of Naval Medical University (Shanghai Changzheng Hospital), Shanghai, China; 2 The Second Affiliated Hospital of Soochow University, Suzhou, Jiangsu, China; 3 Zibo Municipal Hospital, Zibo, Shandong, China; 4 Central Hospital of Dalian University of Technology (Dalian Municipal Central Hospital), Dalian, China

**Keywords:** clinical translation, enzyme resistance, hydrogel, immunomodulation, multifunctional materials, postoperative pancreatic fistula, tissue adhesion

## Abstract

Postoperative pancreatic fistula (POPF) remains one of the most common and potentially lethal complications following pancreatic surgery, with reported incidence rates ranging from 20% to 64%. Conventional sealing materials—including fibrin glue and polyglycolic acid felt—face rapid degradation in the protease-rich pancreatic microenvironment and loss of adhesion in wet conditions, resulting in a marked contradiction between early positive randomized trials and subsequent negative meta-analyses. In recent years, multifunctional hydrogels have been driving a conceptual transition from “passive physical barriers” to “active microenvironment remodeling” for POPF prevention. This review examines hydrogel design strategies tailored to the extreme postoperative pancreatic microenvironment across multiple dimensions: in the domain of enzyme management, we compare the active enzyme-capturing strategy based on D-amino acid peptides, the enzyme-cleavage-site-elimination strategy using recombinant collagen, and the structural tolerance strategy of self-assembling peptides; in structural-functional integration, we analyze how asymmetric bilayer designs simultaneously achieve wet tissue sealing and anti-adhesion; in functional integration, we delineate the evolutionary trajectory of PVA-based platforms from pure physical barriers to antibacterial/anti-inflammatory/pro-healing multifunctional composites; and in the temporal dimension, we evaluate innovative therapeutic frameworks including “full-cycle management” and dual-functional integration of POPF prevention with tumor immunotherapy. We further provide a critical analysis of key translational gaps from five perspectives: species disparities in animal models, quantitative matching of degradation kinetics with anastomotic healing windows, surgical handlability, scalable manufacturing and sterilization, and the diagnostic-therapeutic closed loop. Among the studies reviewed, all therapeutic multifunctional hydrogels remain at the proof-of-concept stage, predominantly in rat models, with SmartPAN—a diagnostic leak indicator—being the only POPF-related hydrogel device to have entered human trials. Establishing standardized pancreatic juice tolerance assessment systems, validating long-term efficacy and safety in large animal models, and achieving precise quantitative matching between degradation kinetics and anastomotic healing timelines represent the most urgent tasks for advancing clinical translation in this field.

## 1 Introduction

### 1.1 Clinical background: epidemiological burden and pathological nature of POPF

Postoperative pancreatic fistula (POPF) is a pathological condition in which pancreatic parenchymal and/or ductal disruption following pancreatic surgery results in abnormal leakage of enzyme-rich pancreatic fluid from the pancreatic remnant or anastomotic site (Shen et al., 2025). As the most common and potentially lethal complication following pancreaticoduodenectomy (PD) and distal pancreatectomy (DP), POPF exhibits considerable variability in reported incidence across the literature: overall rates range from 20% to 64% (Mikamori et al., 2022; Zhou et al., 2026), DP-related POPF is reported at 24.6%–33.2% (She et al., 2026), PD-related POPF may affect up to 30% of patients (Zhou et al., 2026), and in some series, fistula rates can reach as high as 50% (Pausch et al., 2025; Hackert et al., 2016). The fistula risk score (FRS), a prospectively validated clinical tool incorporating pancreatic texture, duct diameter, pathology, and blood loss, accurately predicts POPF risk after PD (Callery et al., 2013), with external validation confirming its discriminative performance across multiple international cohorts (Miller et al., 2014; Roberts et al., 2015).

This variation reflects the evolving definition of POPF—from the International Study Group on Pancreatic Fistula (ISGPF) 2005 definition (Bassi et al., 2005) to the 2016 update (Bassi et al., 2017)—differences in surgical procedures, and the confounding influence of pancreatic texture composition across centers (Nahm et al., 2018; Hartwig et al., 2011). The burden of POPF differs between PD and DP, with distinct risk factor profiles for each procedure (McMillan et al., 2016). Risk factors for DP-related POPF include pancreatic thickness, transection at the body/tail junction, and the absence of selective duct ligation (Ecker et al., 2019; Nathan et al., 2009; Ferrone et al., 2008). High-volume pancreatic centers have accumulated extensive experience, with several large single-institution series documenting complication patterns across thousands of consecutive cases (Kemmochi et al., 2021; Balcom et al., 2001; Winter et al., 2006; Cameron and He, 2015).

However, regardless of the definition employed, the clinical consequences of POPF are severe: enzyme leakage leads to autodigestion of pancreatic and peripancreatic tissues, potentially resulting in intra-abdominal abscess, hemorrhage, surgical site infection, sepsis, delayed gastric emptying, ileus, and multi-organ failure (Shen et al., 2025; She et al., 2026; Cheng et al., 2017). A novel complication grading system specifically applied to pancreatic surgery captures the full spectrum of POPF-related morbidity (DeOliveira et al., 2006), and root-cause analyses of post-pancreatectomy mortality have identified POPF as the dominant driver of surgical death (Vollmer et al., 2012). Overall morbidity following PD ranges from 30% to 50%, with POPF-related mortality of 0%–5% (Zhou et al., 2025); in severe cases, established pancreatic fistula may carry a mortality rate exceeding 40% (Shen et al., 2025). Even in non-lethal cases, POPF significantly prolongs hospitalization, increases healthcare costs, and may delay the initiation of adjuvant chemotherapy—with potential negative implications for long-term oncological outcomes in pancreatic cancer patients (Wu et al., 2014; Siegel et al., 2017).

Despite advances in surgical technique—including microsurgical anastomosis, mattress sutures, binding pancreaticojejunostomy (Peng et al., 2007), modified Blumgart anastomosis (Fujii et al., 2014; Hirono et al., 2019), and pancreatic duct stenting (Motoi et al., 2012; Pessaux et al., 2011; Dong et al., 2016)—and perioperative management such as somatostatin analogs (Büchler et al., 1992; Gurusamy et al., 2013; Allen et al., 2014; Yeo et al., 2000) and goal-directed fluid therapy at high-volume pancreatic centers (Nahm et al., 2018; Kawaida et al., 2019; Shrikhande et al., 2017), the incidence of POPF has not substantially declined over the past several decades (Zhou et al., 2025). Changes in morbidity after pancreatic resection have been documented (Büchler et al., 2003), but a true reduction in POPF remains elusive. This “plateau” phenomenon suggests that refinements in surgical technique alone may be approaching the ceiling of current strategies, underscoring the urgent need for breakthroughs from the perspectives of materials science and bioengineering (Kemmochi et al., 2021; Zhou et al., 2025; He et al., 2024).

### 1.2 The evidence dilemma of existing sealants

Fibrin glue: Fibrin glue has been employed in pancreatic surgery for nearly 35 years (Adelmeijer et al., 2013). The prospective randomized controlled trial (RCT) by Suzuki et al. represents the seminal evaluation of fibrin glue for DP stump closure—56 patients were randomly assigned to the fibrin glue group (n = 26) and the control group (n = 30), with POPF rates of 15.4% (4/26) and 40.0% (12/30), respectively (P = 0.04) (Suzuki et al., 1995). This trial, which defined POPF as pancreatic drainage persisting beyond 7 days with drain amylase greater than three times the serum level, provided preliminary evidence suggesting the potential efficacy of fibrin glue (Suzuki et al., 1995).

However, this trial has clear methodological limitations: single-center design, only 56 patients, and absence of blinding (Suzuki et al., 1995). More critically, subsequent larger-scale meta-analyses have reported no significant benefit of fibrin glue in pancreatic surgery (Kemmochi et al., 2021; Orci et al., 2014). A Cochrane systematic review has provided the most comprehensive evidence synthesis on fibrin sealants for POPF prevention (Gong et al., 2018). An earlier prospective randomized study by D'Andrea et al. likewise failed to demonstrate a clear benefit of human fibrin sealant in preventing pancreatic fistulas (D'Andrea et al., 1994). Lillemoe et al. evaluated fibrin glue sealant application during PD in a prospective randomized trial and similarly found no significant reduction in POPF rates (Lillemoe et al., 2004). Suc et al. reported in a multicenter prospective randomized trial that temporary main pancreatic duct occlusion with fibrin glue did not reduce intra-abdominal complications following pancreatic resection (Suc et al., 2003). Martin and Au also found no decrease in anastomotic leak rates after PD with fibrin glue sealant in their prospective randomized trial (Martin and Au, 2013). This contradiction between an early positive RCT and subsequent negative meta-analyses and trials constitutes a central puzzle in the clinical translation landscape (Kemmochi et al., 2021; Adelmeijer et al., 2013; Orci et al., 2014).

Additional clinical studies further reinforce this conclusion. Montorsi et al. conducted a multicenter RCT evaluating the absorbable fibrin sealant patch (TachoSil) after DP and reported its efficacy (Montorsi et al., 2012), yet subsequent studies yielded mixed results. A systematic review and meta-analysis specifically examined the effectiveness of TachoSil in POPF prevention after DP (Hüttner et al., 2016). Pavlik et al. (2011) and Carter et al. (2013) investigated TachoSil and falciform patch reinforcement respectively, with variable outcomes. Conaglen and Collier (2014) evaluated fibrin glue augmentation during the Whipple procedure (Conaglen and Collier, 2014). Nanashima et al. examined whether fibrin glue prevents biliary and pancreatic fistula after surgical resection (Nanashima et al., 2012). The systematic review and meta-analysis by Orci et al. provided a comprehensive critical appraisal of fibrin sealants in pancreatic surgery (Orci et al., 2014). Collectively, these studies converge on the conclusion that protein-based sealants are insufficient in the pancreatic microenvironment (Kemmochi et al., 2021; Adelmeijer et al., 2013).

Mechanistic explanation—enzymatic degradation: The *in vitro* experiments by Adelmeijer et al., published in the British Journal of Surgery in 2013, provided the critical mechanistic evidence to resolve this puzzle (Adelmeijer et al., 2013). The study directly exposed two liquid fibrin sealants (Tissucol Duo and Evicel) and a carrier-bound fibrin sealant patch (TachoSil) to human pancreatic juice obtained from surgical specimens with ethical approval and informed consent. Liquid sealants exposed to pancreatic juice for 24 h released D-dimer at approximately 1,700 μg/mL, compared to less than 20 μg/mL in the saline control—an 85-fold difference. TachoSil (fibrin plus collagen carrier) was similarly affected: D-dimer at 6 h of pancreatic juice exposure reached 850 ng/mL (versus 60 ng/mL in saline), and hydroxyproline (a collagen degradation marker) at 24 h was 497 μg/mL (versus 26 μg/mL in saline). Serine protease inhibitors (aprotinin, nafamostat mesylate) effectively inhibited degradation—liquid sealant D-dimer dropped to below 50 μg/mL at 24 h, and TachoSil D-dimer to 179 ng/mL at 6 h—but high concentrations of inhibitors simultaneously blocked thrombin activity, thereby preventing fibrinogen-to-fibrin conversion, the very process essential for sealant function (Adelmeijer et al., 2013).

The study concluded that “the use of these products in pancreatic surgery with the aim of preventing leakage of pancreatic fluid is not supported by the present experimental study” (Adelmeijer et al., 2013).

This conclusion not only explains the biological basis for the poor clinical performance of fibrin glue but also establishes a fundamental principle: any sealant with a native protein backbone (fibrin, collagen, gelatin) will rapidly fail in the protease-rich pancreatic microenvironment in the absence of specific enzyme-resistance strategies. This principle has directly shaped the core logic of subsequent hydrogel design—enzyme resistance is not an optional performance enhancement, but rather a prerequisite for material “survival” in the pancreatic microenvironment.

PGA felt (Neoveil): PGA non-woven felt has been applied clinically as an absorbable reinforcement material to the pancreatic stump (Kemmochi et al., 2021; Satoi et al., 2011). Kemmochi et al. evaluated it as a comparator in their rat PF model: ascites amylase in the Neoveil group was 730.2 ± 425.2 IU/L—lower than the untreated control (4992.4 ± 5355.7 IU/L), yet significantly higher than the PVA hydrogel sheet group (303.1 ± 240.1 IU/L, P < 0.05) (Kemmochi et al., 2021). Clinically, PGA felt has likewise “yet to bring about significant improvement” (Kemmochi et al., 2021). Satoi et al. reported reinforcement of pancreaticojejunostomy using PGA mesh and fibrin glue sealant (Satoi et al., 2011), and Akita et al. described a closure method for thick pancreas stump using soft coagulation and PGA felt with fibrin glue (Akita et al., 2015). The ISGPS position statement by Shrikhande et al. further reviewed the evidence for pancreatic anastomotic reinforcement (Shrikhande et al., 2017).

Pancreatic stump closure methods: Beyond sealants, various mechanical closure approaches for DP have been evaluated. The DISPACT multicenter RCT demonstrated no significant difference between stapler and hand-sewn closure (Diener et al., 2011), a finding subsequently supported by meta-analytic evidence (Zhou et al., 2010; Zhang et al., 2015). Specific stapler configurations, including reinforced and triple-row staplers, have been assessed in large observational cohorts (Kawaida et al., 2019; Kawai et al., 2017; Kawaida et al., 2018). However, no closure technique has definitively eliminated POPF, with several single-center series confirming that stapler use alone does not reliably reduce fistula rates (Sugimoto et al., 2013; Kleeff et al., 2007).

Summary of fundamental deficiencies: Conventional sealants share a triple deficiency: (1) absence of controlled degradation—either excessively rapid degradation in protease-rich environments (fibrin glue) or degradation kinetics mismatched with healing progression (Kemmochi et al., 2021; Adelmeijer et al., 2013); (2) lack of intrinsic antibacterial activity—incapable of addressing the risk of postoperative intra-abdominal infection (Zhou et al., 2026); and (3) inadequate mechanical strength and adhesion durability—in dynamic moist environments, unable to maintain effective interfacial sealing (She et al., 2026; Zhang et al., 2022; Li et al., 2017).

### 1.3 Conceptual transition: hydrogels from passive barriers to active microenvironment remodeling

Hydrogels—polymeric materials with three-dimensional hydrophilic network structures—have emerged as promising platforms for postoperative management owing to their tunable mechanical properties, tissue-like compliance conferred by high water content, and sustained therapeutic delivery capacity (Zhou et al., 2026; She et al., 2026; Wang et al., 2025; Yuk et al., 2022). The broader hydrogel field has seen advances ranging from pH-responsive oral delivery systems for gastroprotective agents (Xu et al., 2023a; Zhang et al., 2022) to sustained-release nanoparticle-hydrogel platforms for non-opioid analgesics (Xu et al., 2023b)—each illustrating different aspects of stimuli-responsive design that share conceptual principles with the challenges faced in the pancreatic microenvironment. However, POPF prevention imposes design requirements on hydrogels that far exceed those of general tissue engineering applications. Hydrogels must not only provide sufficient mechanical strength within a range approximating soft tissue mechanics, but must also simultaneously achieve and maintain multiple, sometimes conflicting, functions—strong wet adhesion, enzyme-resistant degradation, antibacterial activity, anti-inflammatory effects, pro-healing properties, and anti-adhesion—under the quadruple extreme conditions of a protease “storm,” alkaline pH, persistent tissue exudate, and potential bacterial contamination (Shen et al., 2025; Zhou et al., 2026; She et al., 2026; Kemmochi et al., 2021; Zhou et al., 2025; He et al., 2024). pH-responsive *in situ* hydrogel systems, such as polyaspartic acid-stabilized CaCO_3_-containing hydrogels for gastric ulcer protection, demonstrate how acid-triggered gelation can be exploited for site-specific barrier formation (Ellis-Behnke et al., 2006)—a design principle that carries conceptual relevance for the alkaline pancreatic environment.

The developmental trajectory of this field clearly reflects a progressive deepening in the understanding of these challenges: from early single-function physical barriers (Kemmochi et al., 2021; Zhao et al., 2024) through active enzyme regulation (He et al., 2024), to multifunctional integration and full-cycle temporal management (Shen et al., 2025; She et al., 2026; Zhou et al., 2025; Pausch et al., 2022).

In what this review terms the Physical Barrier Phase, Kemmochi et al. (2021) first reported the application of a PVA hydrogel sheet in a rat PF model—the sheet remained in place at 48 h postoperatively, with ascites amylase (303.1 vs. control 4992.4 IU/L) and lipase (86.8 vs. 2279.8 IU/L) both significantly reduced. This study provided the first proof of feasibility for hydrogel-based PF prevention, but its design objective was limited to physical sealing (Kemmochi et al., 2021). Mikamori et al. (2022) subsequently evaluated the self-assembling peptide hydrogel SPG-178—ascites amylase at postoperative day 3 was significantly lower than in the untreated group, and hydrogel stiffness remained unchanged following short-duration protease exposure (Mikamori et al., 2022). Earlier foundational work on self-assembling peptide hemostats had established the basis for this class of materials (Zhao et al., 2024).

In what this review terms the Active Enzyme Regulation Phase, the D-amino acid peptide supramolecular hydrogel (CP-CNDS) reported by He et al. in Nature Communications in 2024 (He et al., 2024) marked the inflection point of this conceptual transition—this hydrogel not only passively resists enzymatic degradation but actively captures and sequesters digestive enzymes, interrupting the tissue autodigestion cascade at its source. In the rat POPF model, the CP-CNDS group recovered within merely 3 days, whereas the mock-treated group survived less than 5 days (He et al., 2024).

In what this review terms the Multifunctional Integration and Full-Cycle Management Phase (2024–2025), a series of recent studies have advanced multifunctional hydrogels toward systematic design: Shen et al. (2025) proposed the “full-cycle PF management” framework in Advanced Materials—from Day 1 hemostatic sealing, Days 2–5 anti-inflammatory intervention, Day 3 reversible fibrosis induction, to greater than Day 5 tissue repair—orchestrating the entire PF healing process through temporally-phased regulation via a photoactivated recombinant collagen hydrogel; She et al. (2026) achieved simultaneous strong wet adhesive sealing toward the pancreatic remnant and anti-adhesion protection toward the peritoneal cavity through an asymmetric bilayer design (Ceffe@BH), with eight inflammatory markers restored to normal levels; Zhou et al. (2025) explicitly correlated the degradation kinetics of a PVA-based platform (approximately 60% mass retention at 14 days) with the healing timeline of pancreaticoenteric anastomosis; and Zhao et al. (Pausch et al., 2022) pioneered the integration of POPF prevention and postoperative pancreatic cancer immunotherapy within a single hydrogel platform.

### 1.4 Scope, incremental contributions, and literature selection rationale

This review examines the design strategies of multifunctional hydrogels tailored to the unique postoperative pancreatic microenvironment, focusing specifically on hydrogel-based materials that possess at least one functional feature beyond pure physical sealing—including enzyme resistance/capture, intrinsic antibacterial activity, anti-inflammatory modulation, temporally-phased multi-stage management, or pro-healing bioactivity. In contrast to previous reviews organized by material category, this review adopts a logic chain of “microenvironmental challenges → material design principles → biological effects → translational feasibility,” integrating multiple material strategies for horizontal comparison at each step, and critically analyzing the key gaps between current evidence levels and clinical translation.

Compared with published reviews on medical adhesives and hydrogels—including the systematic review by Wang et al. on hydrogel delivery systems for pancreatic cancer therapy (Wang et al., 2025), the review by Nahm et al. on traditional and emerging concepts in POPF (Nahm et al., 2018), and the review by Kawaida et al. on surgical techniques and postoperative management to prevent POPF (Kawaida et al., 2019)—the incremental contributions of this review lie in the following four aspects: (1) it organizes POPF hydrogel literature through the closed-loop logic of “microenvironmental constraints → design strategy responses → translational gaps,” rather than presenting a literature compilation organized by material category or chronological order; (2) it proposes a tripartite classification of strategies—enzyme capture versus enzyme evasion versus structural tolerance—providing a unified analytical framework for understanding the design philosophy differences underlying distinct anti-enzymatic mechanisms; (3) it establishes “degradation-healing window matching” and “full-cycle management” as core evaluative dimensions, advancing the field’s conceptual shift from “single-function optimization” to “spatiotemporal co-design”; and (4) it introduces the clinical translational experience of SmartPAN intraoperative leak detection (Pausch et al., 2025; Yamashita et al., 2013) into the discussion of therapeutic hydrogels, revealing the diagnostic-therapeutic closed loop as a neglected complementary dimension.

The literature selection focuses on studies employing hydrogels as the primary material platform with POPF prevention as the core objective. The included original research and key clinical studies span a range of surgical, materials science, and translational journals. The SmartPAN series (Pausch et al., 2025; Yamashita et al., 2013) is included as a methodological reference for translational pathways, and the review by Wang et al. (Wang et al., 2025) in Materials Today Bio on hydrogel delivery systems for pancreatic cancer is incorporated as a cross-domain comparative framework for translational challenges. It should be explicitly noted that the original context of Wang et al.’s review (Wang et al., 2025) is pancreatic cancer therapeutic hydrogels; its applicability to the POPF domain is inferential in nature and is transparently indicated as such throughout the text.

## 2 The POPF local microenvironment: constraints on hydrogel “survival” and function

Understanding and quantifying the extreme microenvironmental characteristics of the local POPF milieu constitutes the foundation for rational sealant design. The central thesis of this section is that this microenvironment can be decomposed into four mutually synergistic dimensions—enzymatic activity, wet interface, alkaline pH, and infection/inflammation risk—each imposing quantifiable constraints on specific performance parameters of hydrogels (Shen et al., 2025; Zhou et al., 2026; She et al., 2026; Kemmochi et al., 2021; Zhou et al., 2025; He et al., 2024; Adelmeijer et al., 2013) (Figure 1).

**FIGURE 1:**
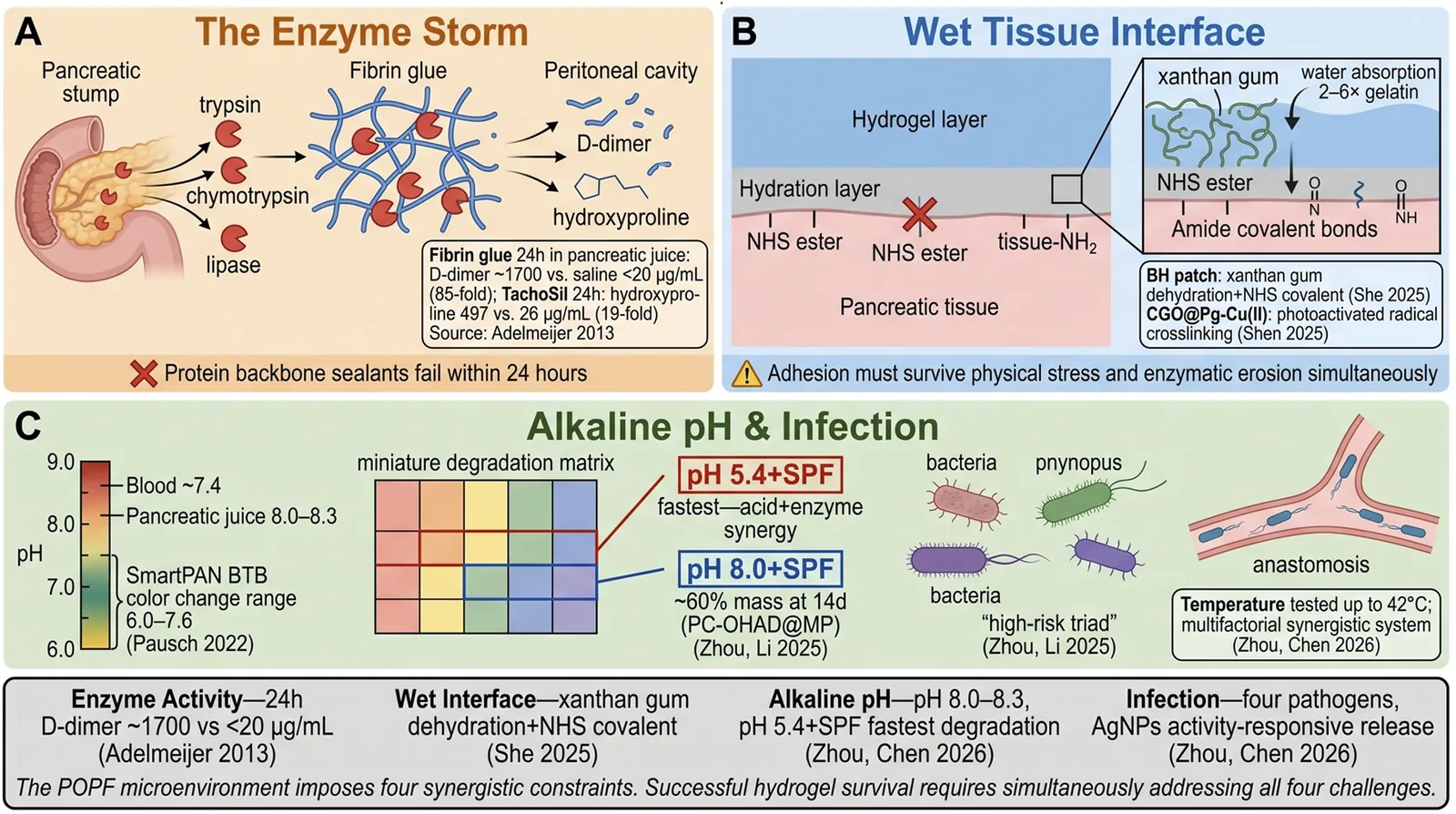
The POPF microenvironment and mechanisms of conventional sealant failure. Panel **(A)** The “enzyme storm”—pancreatic trypsin and chymotrypsin (proteases), together with lipase (a lipolytic enzyme), rapidly degrade fibrin-based sealants. Quantitative data from Adelmeijer et al. (2013) show an 85-fold increase in D-dimer release (liquid fibrin glue, 24 h) and 19-fold increase in hydroxyproline (TachoSil, 24 h) versus saline controls. Panel **(B)** Wet tissue interface—the hydration layer on the pancreatic surface physically blocks covalent bonding of adhesive functional groups to tissue proteins. The BH patch overcomes this via xanthan gum-driven interfacial dehydration combined with NHS ester covalent anchoring (She et al., 2026); CGO@Pg-Cu(II) employs UVA-photoactivated radical crosslinking (Shen et al., 2025). Panel **(C)** Alkaline pH (8.0–8.3) and infection create a multifactorial synergistic microenvironment. The six-condition degradation matrix reveals acid-enzyme synergy as the fastest degradation mode (Zhou et al., 2026). Footnote: The POPF microenvironment imposes four synergistic constraints. Successful hydrogel survival requires simultaneously addressing all four challenges.

### 2.1 The “enzyme storm”: degradation kinetics and failure mechanisms of conventional materials

The digestive enzymes in pancreatic exocrine secretions constitute the primary chemical threat to sealant materials. The major proteases in pancreatic juice are trypsin and chymotrypsin; additionally, lipase—a lipolytic enzyme that hydrolyzes triglycerides—and α-amylase contribute to the broader degradative milieu (He et al., 2024). Upon the occurrence of POPF, these enzymes extravasate from the leakage site into the peritoneal cavity, not only directly digesting the pancreas itself and surrounding tissues—leading to “autodigestion” and subsequent intra-abdominal abscesses and lethal sepsis (Cheng et al., 2017; He et al., 2024)—but also degrading protein-based biomaterials used for sealing and reinforcement with considerable efficiency (Adelmeijer et al., 2013).

The quantitative data from Adelmeijer et al. (2013) (Table 1) serve as the cornerstone for understanding this “material failure.” The 24-h D-dimer release from liquid fibrin sealants in pancreatic juice (approximately 1,700 μg/mL versus saline below 20 μg/mL, an 85-fold difference) signifies near-complete degradation of the sealant’s fibrin backbone within a single day. The results for TachoSil are particularly illustrative—not only was its fibrin component degraded (6-h D-dimer 850 ng/mL), but its collagen carrier was similarly destroyed (24-h hydroxyproline 497 μg/mL)—demonstrating that no protein component can survive in this environment (Adelmeijer et al., 2013).

**TABLE 1:** Degradation data of conventional sealants in pancreatic juice (In vitro).

Sealant type	Exposure medium	Time point	Degradation marker	Value in pancreatic juice	Value in saline	Pancreatic juice/saline ratio	With protease inhibitor	Data source
Liquid fibrin glue (Tissucol Duo/Evicel)	Human pancreatic juice	24 h	D-dimer (μg/mL)	∼1700	<20	∼85	<50	Adelmeijer et al. (2013)
Carrier-bound fibrin patch (TachoSil)	Human pancreatic juice	6 h	D-dimer (ng/mL)	850	60	∼14	179	Adelmeijer et al. (2013)
Carrier-bound fibrin patch (TachoSil)	Human pancreatic juice	24 h	Hydroxyproline (μg/mL)	497	26	∼19	181	Adelmeijer et al. (2013)
Fibrin glue (clinical)	DP patients	Postoperative	POPF rate	—	—	—	—	Suzuki et al. (1995): 15.4% vs. 40.0% (P = 0.04); Subsequent meta-analyses: no significant benefit (Kemmochi et al., 2021; Orci et al., 2014; Gong et al., 2018)

This mechanistic explanation aligns closely with the clinical evidence: the contradiction between the early positive single-center RCT by Suzuki et al. (1995) and the negative findings of subsequent large-scale meta-analyses (Kemmochi et al., 2021; Orci et al., 2014; Gong et al., 2018) likely reflects the substantial heterogeneity in pancreatic juice enzymatic activity across different patient populations and surgical scenarios, as well as the complete failure of fibrin glue under conditions of higher enzymatic activity. As discussed by Adelmeijer et al. (2013), although Tissucol Duo contains the serine protease inhibitor aprotinin, “the concentration is apparently too low to inhibit fibrinolysis elicited by pancreatic juice.”

Implications for hydrogel design: Enzyme resistance is not an optional performance enhancement but rather a necessary condition for hydrogel “survival” in the pancreatic microenvironment. This principle directly informs all design strategies discussed in Section 3.1 below—from stereochemistry to structural evasion to active enzyme capture. Furthermore, it reveals an inadequacy of current *in vitro* evaluation systems: existing *in vitro* enzyme degradation experiments typically employ purified single trypsin species, whereas authentic pancreatic juice represents a complex mixture of multiple enzymes and cofactors—whose degradative capacity may be systematically underestimated (Zhou et al., 2025; He et al., 2024; Adelmeijer et al., 2013).

### 2.2 The physicochemistry of wet tissue interface adhesion

The persistent exudate on the surface of the pancreatic remnant or anastomotic site creates one of the most challenging scenarios in adhesion science—the “wet interface.” She et al. (She et al., 2026) explicitly identified that “the peritoneal fluid microenvironment maintains continuous wetness on tissue surfaces, which severely compromises material adhesion,” and recognized “the moist surgical environment” and “the complex post-pancreatectomy pathophysiology” as two fundamental challenges impeding the clinical translation of hydrogels in POPF management.

The physicochemical mechanism underlying this phenomenon is that the hydration layer on the tissue surface forms a physical barrier at the hydrogel-tissue interface, disrupting the covalent crosslinking between hydrogel functional groups and tissue surface proteins (She et al., 2026; Zhang et al., 2022; Li et al., 2017). Conventional chemical adhesives perform well on dry surfaces but cannot effectively polymerize and anchor on moist, dynamic pancreatic remnants. Fibrin glue, while capable of fibrin polymerization in a wet environment, has adhesion strength inherently limited by non-covalent interactions (hydrogen bonds, van der Waals forces) at the fibrin-tissue interface, and its fibrin backbone is rapidly degraded by pancreatic proteases, as discussed in Section 2.1 (Adelmeijer et al., 2013).

Pancreatic texture itself introduces additional complexity. Shen et al. (Shen et al., 2025) characterized pancreatic tissue as “soft, fragile in texture (resembling tender tofu)” with “extremely poor suture-holding capacity (SHC).” This low SHC implies that conventional strategies relying on sutures for tissue apposition and sealing possess inherent mechanical limitations—the tension applied by sutures generates significant shear stress within the fragile pancreatic parenchyma, potentially causing tissue tearing that paradoxically triggers POPF (She et al., 2026). Furthermore, the pancreatic remnant undergoes dynamic postoperative edema (She et al., 2026), and the sustained shear effect of sutures may further exacerbate tissue injury.

Wüster et al. reported that abnormal activation of pancreatic enzymes upon contact with blood or pancreatic duct ligation obstruction represents another major factor contributing to POPF [as cited in 4]. Enzyme activation at the pancreatic resection margin not only triggers tissue autodigestion but also degrades the protein components of the adhesion interface—meaning that the adhesion interface must withstand not only physical stress but also sustained chemical erosion.

Comprehensive requirements for hydrogel adhesion design: (1) the ability to penetrate or displace the interfacial hydration layer (e.g., the dehydration strategy of xanthan gum) (She et al., 2026); (2) the formation of stable interfacial bonds that transcend hydrogen bonding (e.g., covalent bonds via NHS esters, coordination bonds via dopamine, photoactivated radical crosslinking) (Shen et al., 2026; She et al., 2026; Zhang et al., 2022); (3) the maintenance of bond stability in an enzymatically active environment (i.e., the adhesion interface itself also requires anti-enzymatic protection) (She et al., 2026; Adelmeijer et al., 2013); and (4) mechanical properties that conform to, rather than cut through, fragile pancreatic tissue (Shen et al., 2025; She et al., 2026). The BH patch discussed in Section 3.2 (She et al., 2026) and CGO@Pg-Cu(II) discussed in Section 3.4.1 (Shen et al., 2026) address these comprehensive requirements to varying degrees.

### 2.3 Alkaline pH and the dynamic physicochemical microenvironment

The physiological pH of pancreatic juice is 8.0–8.3—this is the chemical basis enabling SmartPAN, a hydrogel indicator employing bromothymol blue (BTB) as the active pH indicator and polysaccharide microspheres as the matrix, to achieve intraoperative leak detection (Pausch et al., 2025; Yamashita et al., 2013; Diamond and Freeman, 2001; Mori et al., 2015). BTB has a pKa of approximately 7.0 and undergoes a color transition from yellow through green to blue within the pH range of approximately 6.0–7.6. At blood pH (∼7.4), BTB appears greenish—close to the transition midpoint—whereas at pancreatic juice pH (8.0–8.3), it shifts definitively to blue, enabling visual discrimination between blood contamination and pancreatic fluid leakage (Pausch et al., 2025). It should be noted that the practical reliability of this colorimetric discrimination depends on sufficient pH differential; in clinical scenarios where blood and pancreatic fluid mix, visual interpretation may be confounded by intermediate color states, a limitation acknowledged by the ViP trial (Pausch et al., 2025).

This alkaline microenvironment significantly impacts hydrogel degradation kinetics. Zhou et al. (2025), in their degradation study of the PC-OHAD@MP hydrogel, specifically simulated “the special pH environment (pH 7.15–8.93) of the chronic wound healing phase around pancreaticoduodenal anastomosis.” Results demonstrated that PVA hydrogel degradation rates were markedly higher under simulated pancreatic fluid conditions (pH 8.0) than under other pH conditions (Zhou et al., 2025). Zhou et al. (2026), in a more systematic six-condition degradation experiment (pH 5.4/7.4/8.0 × PBS/SPF), observed that degradation rates in enzyme-containing simulated pancreatic fluid (SPF) far exceeded those in PBS at the same pH, and that the combination of acidic pH (5.4) with SPF exhibited the fastest mass loss—“indicating a synergistic effect between acid-catalyzed hydrolysis and enzymatic cleavage” (Zhou et al., 2026).

Furthermore, although precise local temperature changes *in vivo* have not been directly measured in POPF models, Zhou et al. (2025) incorporated elevated temperature conditions (up to 42 °C) into their *in vitro* degradation experiments to model the elevated tissue temperature that may accompany postoperative inflammation—suggesting the potential relevance of temperature as a degradation-accelerating factor, though *in vivo* validation data are currently lacking.

Collectively, these findings demonstrate that the local POPF microenvironment constitutes a multifactorial synergistic dynamic system—pH, temperature, enzymatic activity, and inflammatory mediators interact reciprocally, such that changes in any single factor may alter the overall degradation behavior of hydrogels through cascading effects (Zhou et al., 2026; Zhou et al., 2025).

Implications for hydrogel design: Degradation testing must be performed under multi-factorial conditions that simulate the authentic pancreatic microenvironment, rather than solely in standard PBS. The six-condition degradation matrix of Zhou et al. (2026) and the multi-pH/multi-temperature testing system of Zhou et al. (2025) provide methodological exemplars for this approach. Moreover, the evaluation of degradation kinetics must incorporate the temporal dimension—it is necessary to understand not only “how fast degradation occurs” but also “how the degradation rate changes across different postoperative phases”—because the microenvironment itself (pH, enzymatic activity, temperature) is dynamically evolving over time (Zhou et al., 2026; Zhou et al., 2025).

### 2.4 Infection risk, microbial spectrum, and antibacterial requirements

Once POPF occurs, the autodigestion of surrounding tissues by pancreatic enzymes creates an ideal culture medium for bacterial colonization (Shen et al., 2025; Cheng et al., 2017; Zhou et al., 2025). “Severe pancreatic fistula can lead to abdominal infection and hemorrhage, even progressing to severe sepsis” (Zhou et al., 2025). Microbial colonization is recognized as one of the key drivers of postoperative complications (Zhou et al., 2026).

From anatomical and physiological perspectives, the pancreaticoenteric anastomosis (in the PD procedure) faces a distinctive infection risk: this site is simultaneously exposed to high digestive enzyme activity, potential microbial contamination from the intestinal lumen (via anastomotic microleakage), and tissue mechanical stress, together constituting a “high-risk triad” (Zhou et al., 2025). Zhou et al. (2025) specifically evaluated the antibacterial activity of their PC-OHAD@MP hydrogel against four common pathogenic bacteria: *Staphylococcus aureus*, *Escherichia coli*, *Pseudomonas aeruginosa*, and *Klebsiella* pneumoniae—covering the major representative species of Gram-positive cocci, Gram-negative bacilli, and non-fermenting bacteria encountered in intra-abdominal infections. Zhou et al. (2026) similarly validated the broad-spectrum antibacterial activity of their PVA/QCS/AgNP hydrogel against pathogens including *S. aureus* and *E. coli*.

Implications for hydrogel design: Physical sealing alone is far from sufficient to prevent POPF-related infectious complications. An ideal POPF-preventive hydrogel should provide effective local antibacterial protection during the early postoperative period (the window of highest infection risk) to prevent bacterial colonization and biofilm formation along the anastomotic line or remnant surface (Zhou et al., 2026; Cheng et al., 2017; Zhou et al., 2025). Conventional fibrin glue and PGA felt precisely lack this critical function—Zhou, Chen, et al. (Zhou et al., 2026) explicitly noted that these materials “lack inherent antibacterial properties and are ineffective against microbial colonization.” This requirement has directly driven the integration of antibacterial AgNPs (Zhou et al., 2026), the antibiotic meropenem (Zhou et al., 2025), and penicillin G-Cu(II) nanodrugs (Shen et al., 2025), as discussed in Section 3.3. However, it should be acknowledged that antibacterial functionality is more critical for certain clinical scenarios (e.g., PD with intestinal contamination, reoperative surgery, immunocompromised patients) than for others (e.g., straightforward DP with a clean surgical field), and should not be imposed as a universal requirement for all sealant designs without considering the clinical context.

## 3 Design strategies for multifunctional hydrogels: from molecular mechanisms to biological effects

This chapter is organized around the sequential pathophysiological cascade following POPF onset—sealing leakage → inhibiting enzymatic activity → controlling infection → modulating inflammation → promoting tissue repair → preventing adhesions—addressing, in turn, the hydrogel design strategies developed for each stage (Figure 2). The discussion of each strategy covers design rationale, key performance data, biological effect evidence, and evidence level assessment. Each strategy section concludes with a “Comparison and Synthesis” subsection, conducting horizontal comparison based on unified performance dimensions. Direct quantitative comparisons of efficacy across studies are precluded by substantial heterogeneity in animal models, PF induction methods, observation periods, and endpoint definitions; where numerical data are cited, the specific testing conditions and endpoints of each study should be carefully noted.

**FIGURE 2:**
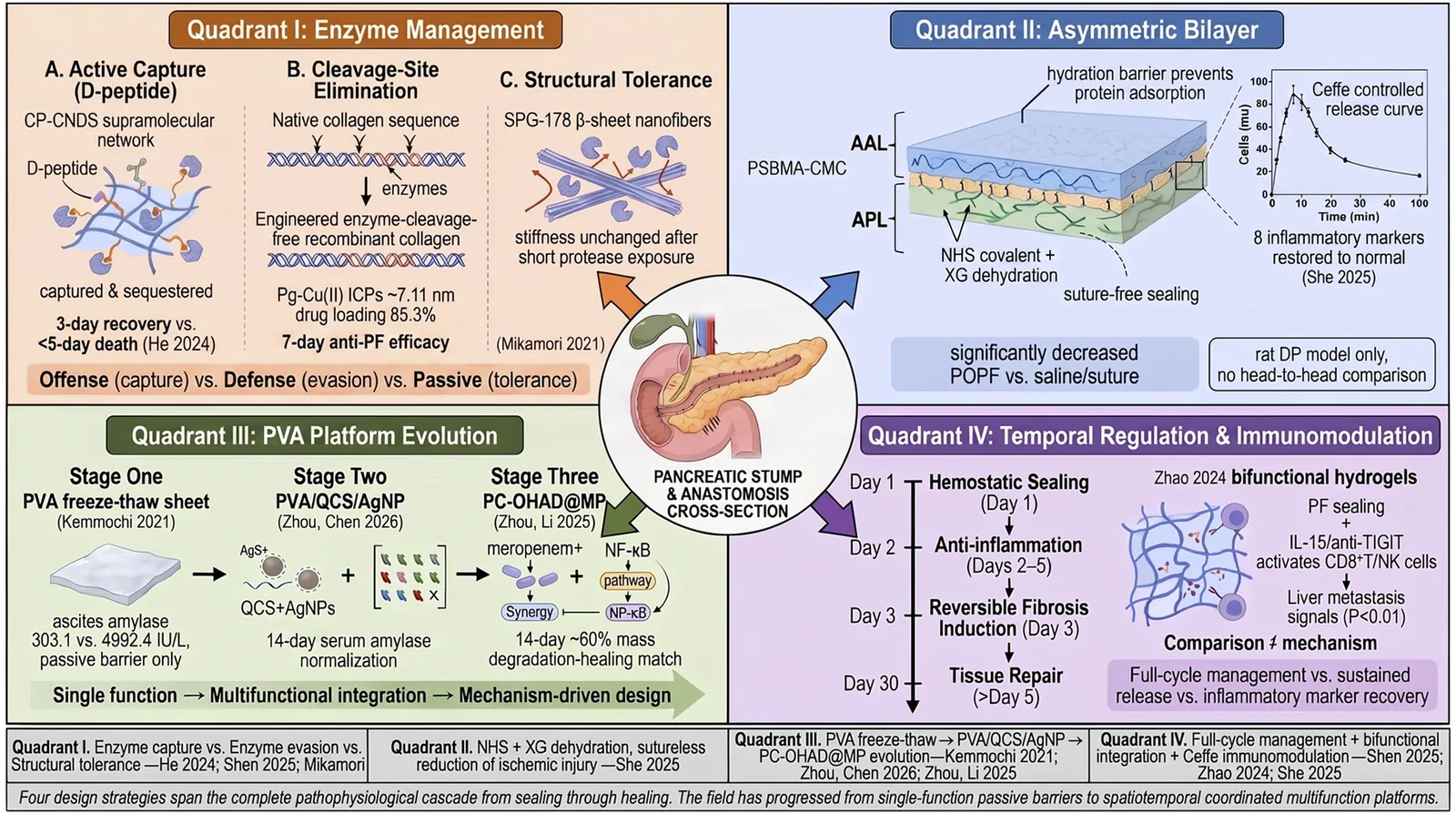
Design strategies for multifunctional POPF hydrogels: a panoramic overview. Quadrant I (Enzyme Management): Three strategies—active enzyme capture via D-peptide supramolecular hydrogel CP-CNDS (He et al., 2024), cleavage-site elimination in recombinant collagen CGO@Pg-Cu(II) (Shen et al., 2025), and structural tolerance via β-sheet self-assembling peptide SPG-178 (Mikamori et al., 2022). Quadrant II (Asymmetric Bilayer Design): Ceffe@BH dual-layer patch with NHS/xanthan gum adhesive pancreatic layer and zwitterionic anti-adhesion layer; eight inflammatory markers restored to normal levels (She et al., 2026). Quadrant III (PVA Platform Evolution): Progression from pure PVA freeze-thaw sheet (Kemmochi et al., 2021) to PVA/QCS/AgNP dual-crosslinked antibacterial system (Zhou et al., 2026) to PC-OHAD@MP with targeted antibacterial, NF-κB anti-inflammatory modulation, and degradation-healing matching (Zhou et al., 2025). Quadrant IV (Temporal Regulation and Immunomodulation): Full-cycle PF management with four temporally-defined phases (Shen et al., 2025) and dual-functional POPF prevention with tumor immunotherapy via IL-15/anti-TIGIT (Zhao et al., 2024). Footnote: These four strategy domains span the complete pathophysiological cascade from sealing through healing.

### 3.1 Strategy I: enzyme resistance and enzyme capture—molecular strategies to interrupt the autodigestion cascade

As established in Section 2.1, addressing the digestive enzymes in pancreatic fluid constitutes the foremost dimension of hydrogel design—this is not an optional performance enhancement but rather a prerequisite for material “survival” in this microenvironment (He et al., 2024; Adelmeijer et al., 2013). Based on the molecular mechanisms of action, the strategies reported in the published literature can be classified into three categories: stereochemical strategy (enzyme capture based on D-amino acids), structural evasion strategy (recombinant protein design with eliminated cleavage sites), and structural tolerance strategy (physical stability based on β-sheet nanofibers) (Shen et al., 2025; Mikamori et al., 2022; He et al., 2024).

#### 3.1.1 Stereochemical strategy: active enzyme capture and sequestration by D-amino acid peptides

The CP-CNDS (Covalent Peptide-Chiral Network D-peptide Supramolecular gel) reported by He et al. in Nature Communications in 2024 (He et al., 2024) represents a distinctive enzyme-addressing strategy in the published literature. Its core design philosophy involves not the conventional “resistance” to enzymatic degradation (i.e., enhancing the hydrogel’s own enzyme tolerance), but rather the creation of “enzyme coordinators”—actively capturing digestive enzymes from the surrounding solution and irreversibly sequestering them within the hydrogel interior, thereby not only protecting the hydrogel itself but also preventing enzyme-mediated tissue attack at its source (He et al., 2024).

Molecular design rationale: The backbone of CP-CNDS is constructed from the D-amino acid peptide motif Ac-(rada)_4_-NH_2_. Native L-amino acid peptides serve as natural substrates for trypsin and chymotrypsin—these enzymes recognize the specific stereochemical configuration of L-amino acids and specific peptide bonds to effect catalytic hydrolysis. D-amino acid peptides, by virtue of the complete inversion of the chiral center (from L to D), fundamentally disrupt the spatial recognition complementarity between enzyme and substrate—trypsin cannot recognize D-peptides as substrates and is therefore incapable of cleavage (He et al., 2024). More critically, the supramolecular network of CP-CNDS, stabilized through multiple non-covalent interactions (including, but not limited to, intermolecular hydrogen bonding, π-π stacking, and hydrophobic effects), forms a stable gel structure whose internal cavities and binding sites can “capture” digestive enzymes from solution and “effectively sequester them, preventing their exchange with enzymes in the external environment,” resulting in the “complete suppression of digestive enzyme activity” (He et al., 2024).

Key biological effects: In a rat POPF model established through surgical creation of a pancreatic fistula based on the method of Tanaka et al. (2013), rats treated with CP-CNDS “not only survived but recovered within merely 3 days,” whereas mock-treated POPF rats “survived less than 5 days while experiencing postoperative pancreatic fistula, leakage, or abscess” (He et al., 2024). The investigators also employed molecular dynamics (MD) simulations to evaluate the binding affinities between the D-peptide motif and trypsin, lipase, and chymotrypsin (He et al., 2024), providing computational support for the molecular mechanism of enzyme capture.

Evidence level assessment: This study constitutes a proof-of-concept animal experiment. Strengths include: (1) originality of design concept—a shift from “passive defense” to “active offense”; (2) multi-level validation—in vitro enzyme activity assays, MD simulations, and survival endpoints in a rat PF model; (3) large effect size—from less than 5-day survival to 3-day recovery. Limitations include: sample size not explicitly reported in the accessible text; long-term safety (the metabolic fate and immunogenicity of D-peptides *in vivo*) not assessed; validation limited to healthy rats; and the enzyme capture capacity and kinetics of CP-CNDS in large animals or humans not characterized (He et al., 2024).

#### 3.1.2 Structural evasion strategy: systematic elimination of cleavage sites in recombinant collagen

The CGO@Pg-Cu(II) hydrogel reported by Shen et al. in Advanced Materials in 2025 (Shen et al., 2025) employs a strategy fundamentally different from that of CP-CNDS. Its anti-enzymatic mechanism does not involve active enzyme capture but rather the elimination of cleavage sites at the root of molecular design—employing glycidyl methacrylate-modified recombinant collagen that is “enzyme-cleavage-free” as the backbone molecule (Shen et al., 2025).

Design logic: Native collagen contains multiple recognition and cleavage sites for trypsin and collagenase (e.g., Arg-Gly and Lys-Gly sequences), and would therefore undergo rapid degradation in the pancreatic microenvironment (Shen et al., 2025; Adelmeijer et al., 2013). The recombinant collagen used by Shen et al. (2025) was engineered, at the design stage, to systematically remove or replace these cleavage sites through sequence engineering, rendering it structurally “unrecognizable”—a design philosophy that parallels the covalent peptide hydrogel strategy reported by the Kimmins group [as cited in 1]: both approaches regulate enzymatic degradation behavior through the systematic manipulation of peptide/protein amino acid composition and stereochemistry.

Integration with functional nanodrugs: Another key innovation of CGO@Pg-Cu(II) lies in combining the enzyme-resistant backbone with functional nanodrugs—penicillin G-Cu(II) infinite coordination polymers (Pg-Cu(II) ICPs). The Pg-Cu(II) ICPs are formed through multidentate coordination between Cu(II) and penicillin G, yielding spherical particles of approximately 7.11 ± 0.25 nm (Shen et al., 2025), with a drug loading capacity of 85.3% and encapsulation efficiency of 97.8%. The incorporation of Cu(II) serves a dual purpose: (1) intrinsic inhibitory activity against trypsin; and (2) conferring pH-responsiveness to the nanodrugs—the coordination polymers dissociate within the acidic pH range of 4.5–6.3 (Shen et al., 2025), ensuring that Pg-Cu(II) ICPs remain stable under the alkaline conditions of PF while effectively releasing penicillin G upon cellular internalization into the acidic lysosomal environment. This design effectively addresses the multiple delivery barriers of penicillin G (water solubility as high as 330 mg/mL, *in vivo* half-life of merely 30–60 min, and difficulty in passive diffusion across the lipid bilayer of mammalian cells due to the absence of hydrophobic moieties) (Shen et al., 2025).

Evidence level: Comparable to that of CP-CNDS—a proof-of-concept animal experiment (rat PF model), though CGO@Pg-Cu(II) provides a more detailed theoretical framework (full-cycle management, see Section 3.4.1) and more systematic material characterization (TEM particle size, drug loading, encapsulation efficiency, pH-responsive release) (Shen et al., 2025).

#### 3.1.3 Structural tolerance strategy: physical stability of β-sheet self-assembling peptides

The SPG-178 reported by Mikamori et al. (2022) represents an earlier, physically rather than chemically based approach to enzyme tolerance. SPG-178 has the amino acid sequence [CH_3_CONH]-RLDLRLALRLDLR-[CONH_2_] and self-assembles into a nanofiber network in aqueous solution through the formation of stable β-sheet secondary structures (Mikamori et al., 2022).

Mechanism of enzyme tolerance: The β-sheet assemblies of SPG-178 arrange hydrophobic residues (Leu) and hydrophilic residues (Arg, Asp) into highly ordered nanofiber architectures. This tightly packed structure may restrict the accessibility of enzyme molecules to potential cleavage sites (steric hindrance), thereby conferring a degree of passive enzyme tolerance (Mikamori et al., 2022). The key finding of the investigators was that SPG-178 exhibited no change in stiffness following short-duration protease treatment (Mikamori et al., 2022), suggesting that the physical integrity of the β-sheet nanofiber network is preserved under short-term enzyme exposure.

In a rat PF model established by transecting the splenic-side pancreatic duct according to the method of Tanaka et al. (2013), SPG-178 was applied to both sides of the pancreatic remnant, and ascites amylase at postoperative day 3 was significantly lower than in the untreated group; pathological examination revealed fewer inflammatory cells on the surface of the pancreatic remnant in the SPG-178 group and the formation of “a structure composed of fibrous tissue or mesothelium covering it” (Mikamori et al., 2022)—suggesting that the hydrogel served as a scaffold for tissue regeneration.

Evidence level: Proof-of-concept study. Strengths: first demonstration of the feasibility of a self-assembling peptide hydrogel for PF prevention (Mikamori et al., 2022). Limitations: (1) only short-duration protease exposure was tested—long-term stability was not evaluated; (2) the enzyme tolerance of SPG-178 relies on physical structure rather than engineered chemical modification, and its degradation fate under prolonged *in vivo* pancreatic fluid exposure is unknown (Mikamori et al., 2022); (3) assessment was limited to ascites amylase and lipase, lacking multi-dimensional evaluation of inflammation, healing, and tissue remodeling.

#### 3.1.4 Comparison and synthesis: systematic evaluation of three enzyme-addressing strategies

Placing these three strategies within a unified comparative framework reveals their respective design philosophies and trade-offs:

**Table:** 

Comparative Dimension	CP-CNDS (He et al., 2024)	CGO@Pg-Cu(II) (Shen et al., 2025)	SPG-178 (Mikamori et al., 2022)
Core mechanism	Active enzyme capture and sequestration	Systematic elimination of cleavage sites	Physical steric hindrance
Design philosophy	“Offense”—neutralize enzyme activity	“Defense”—avoid recognition as substrate	“Passive defense”—limit enzyme accessibility
Mode of enzyme inhibition	Capture + sequestration (prevent enzyme exchange)	Non-cleavable + Cu(II) trypsin inhibition	Restrict enzyme access to cleavage sites
Tissue protection	Direct—block autodigestion at source	Indirect—physical barrier + fibrotic reinforcement	Indirect—sealing only
Key *in vivo* endpoint	3-day recovery vs. <5-day death	100% anti-PF efficacy at Day 7	Decreased ascites amylase at Day 3
Observation period	∼3–5 days	7 days	3 days
Long-term safety	D-peptide metabolic fate unassessed	Reversibility of fibrosis unverified	Long-term degradation behavior unassessed
Preparation complexity	Supramolecular self-assembly	UVA photocrosslinking (365 nm, 7 mW/cm^2^, 10 s)	Self-assembly

Integrative analysis: These three strategies represent three distinct tiers of enzyme management. The passive structural tolerance of SPG-178 is an early strategy suitable for short-term sealing but unable to cope with sustained enzyme exposure (Mikamori et al., 2022). The active enzyme capture of CP-CNDS is philosophically the most radical—the shift from “passive defense” to “active offense” may achieve more complete suppression of enzyme activity, but the specificity of its D-peptide motifs (whether they exclusively capture pancreatic enzymes without affecting normal coagulation cascades and inflammatory/immune mediators) requires more systematic evaluation (He et al., 2024). The cleavage-site-elimination strategy of CGO@Pg-Cu(II) offers a “cleaner” solution—addressing the problem through design-based exclusion rather than functional capture—though its effectiveness depends on the completeness of cleavage-site elimination and the fidelity of recombinant protein expression (Shen et al., 2025).

The above comparison focuses on design philosophy and conceptual differences at the strategic level; systematic comparison of the specific technical parameters corresponding to each strategy (crosslinking mode, degradation conditions, adhesion strategy, preparation process) is provided in Table 2.

**TABLE 2:** Comparison of physicochemical properties and preparation characteristics of multifunctional POPF hydrogels.

Hydrogel system	Backbone material	Crosslinking mode	Preparation conditions	Key functional component	Adhesion strategy	Enzyme-addressing mechanism	Antibacterial strategy	Degradation conditions (test environment)	Key degradation data	Reference
PVA sheet	PVA	Freeze-thaw	Freeze-thaw cycles	None	Physical coverage/hydrogen bonding	None specifically designed	None	Not reported	Remained in place at 48 h (*in vivo* rat observation)	Kemmochi et al. (2021)
SPG-178	Self-assembling peptide (β-sheet)	Self-assembly	Aqueous self-assembly	None	Physical coverage	Structural tolerance (short-term)	Not reported	Short-term protease exposure (*in vitro*)	Stiffness unchanged upon short-term exposure; long-term not assessed	Mikamori et al. (2022)
CP-CNDS	D-amino acid peptide	Supramolecular self-assembly	Non-covalent interactions	None	Physical coverage	Active enzyme capture + sequestration	Not reported	Not reported	No quantitative degradation data provided	He et al. (2024)
(P-aTIGIT&PDA@IL-15)@gel	MA-HA-DA + SH-HA-DA	Dual crosslinking	Chemical crosslinking	PDA@IL-15 + platelet-anti-TIGIT	Dopamine-based adhesion	PDA protects immunotherapeutic agents	Not specifically designed	Not reported	No quantitative degradation data provided	Zhao et al. (2024)
CGO@Pg-Cu(II)	Recombinant collagen (cleavage-site-free) + oxidized pullulan	UVA photocrosslinking + dynamic imine bonds	365 nm, 7 mW/cm^2^, 10 s; 0.15% I2959; total solids 13.5%; CF-GMA:OP = 2:1	Pg-Cu(II) ICPs (∼7.11 nm, drug loading 85.3%, encapsulation 97.8%)	Photoactivated radical crosslinking to tissue	Systematic cleavage-site elimination + Cu(II) trypsin inhibition	Penicillin G-Cu(II)	Not reported	No quantitative degradation data provided	Shen et al. (2025)
Ceffe@BH (bilayer)	APL: PSBMA + AAc-NHS + AAc + XG; AAL: PSBMA-CMC	UV polymerization (APL second network)	UV polymerization (wavelength/power/duration not reported)	Ceffe (growth factors + exosomes + ECM proteins)	NHS covalent bonding + xanthan gum dehydration	XG enzyme resistance + covalent bonding stability	Present (MIC values not reported)	Not reported	No quantitative degradation data provided; original text reports “prolonged post-swelling adhesion retention and enzymatic resistance”	She et al. (2026)
PC-OHAD@MP	PVA + CS + OHAD	Freeze-thaw (3 cycles) + dynamic bonds	−20 °C/20 h, 25 °C/4 h; PVA 10 wt%; OHAD/CS/PVA = 1:1:2	Meropenem (0.17 mg/mL)	Dopamine-based adhesion	Accelerated but controllable in simulated pancreatic fluid (pH 8.0)	Meropenem (*S. aureus*, *E. coli*, *P. aeruginosa*, *K. pneumoniae*)	pH 8.0 SPF; 25/37/42 °C	14 days–60% mass retention; continued degradation >20 days	Zhou et al. (2025)
PVA/QCS/AgNP	PVA + QCS	UV-reduced Ag^+^ + freeze-thaw	UV + freeze-thaw cycles	QCS + AgNPs	Physical coverage	Material-level enzyme resistance	QCS membrane disruption + Ag^+^ oxidative stress	Six-condition matrix (pH 5.4/7.4/8.0 × PBS/SPF); 37 °C	pH 5.4+SPF fastest degradation (synergy); specific values per condition in original figures	Zhou et al. (2026)

Hypothesis (to be validated): A hybrid hydrogel combining enzyme capture capability (e.g., D-peptide motifs) and enzyme evasion capability (e.g., recombinant collagen) may, through synergistic complementarity—enzyme capture providing immediate protection and the enzyme-evading backbone providing long-term stability—achieve sealing durability surpassing that of either single strategy. This hypothesis requires validation through *in vitro* pancreatic fluid degradation experiments and long-term *in vivo* follow-up.

### 3.2 Strategy II: asymmetric bilayer design—functional zoning of tissue sealing and anti-adhesion

Postoperative adhesive intestinal obstruction is a common long-term complication following DP, with its pathological mechanism involving an inflammatory cell-mediated fibrotic process (She et al., 2026; Diamond and Freeman, 2001). This defines a structural dilemma: the side of the hydrogel facing the pancreatic remnant/anastomosis requires maximized adhesion and sealing, while the side facing the peritoneal cavity requires the diametrically opposite properties—resistance to protein adsorption and cell adhesion (She et al., 2026). The asymmetric bilayer hydrogel (BH patch) reported by She et al. (She et al., 2026) is the only solution among the reviewed studies that systematically addresses this contradiction.

#### 3.2.1 Design rationale of the bilayer architecture

Inner layer—adhesive pancreatic layer (APL): This employs a dual-network design. The first network uses polysulfobetaine methacrylate (PSBMA) as the scaffold material; the second network is formed by UV-initiated polymerization after immersion in a composite solution containing acrylic acid N-hydroxysuccinimide ester (AAc-NHS), acrylic acid (AAc), xanthan gum (XG), and Ceffe (She et al., 2026).

The dual adhesion mechanism constitutes the core innovation of the APL. The first mechanism—NHS ester-mediated covalent anchoring: NHS groups form stable amide bonds with primary amine groups (lysine residue side chains) on tissue surface proteins—this represents covalent, irreversible strong adhesion (She et al., 2026). The second mechanism—xanthan gum-driven interfacial dehydration: food-grade xanthan gum (XG), whose water absorption capacity at equal mass concentration exceeds that of gelatin by 2–6 times (She et al., 2026), acts as a “colloidal desiccant” at the hydrogel-tissue interface, effectively depleting the interfacial hydration layer, optimizing interfacial contact, and creating conditions for subsequent NHS covalent crosslinking (She et al., 2026). This combined strategy—dehydration first, covalent crosslinking second—mimics the underwater adhesion principles of marine organisms (mussels and barnacles) (Zhang et al., 2022; Li et al., 2017): enhancing chemical crosslinking by removing interfacial water from the contact interface (She et al., 2026).

Furthermore, xanthan gum, as a macromolecular polysaccharide with a stable double-helical molecular structure, “exhibits potent antioxidant capacity and excellent enzyme resistance,” endowing the hydrogel with “significant protective effects against pancreatic enzyme degradation” (She et al., 2026), ensuring the durable integrity of sealing at the pancreatic resection margin.

Outer layer—anti-adhesion layer (AAL): This is composed of PSBMA-carboxymethyl chitosan (PSC) (She et al., 2026). The sulfobetaine zwitterionic polymer forms a tightly bound hydration layer on its surface through electrostatic induction, creating a potent physical barrier effect that prevents non-specific protein adsorption and subsequent fibroblast adhesion—the initiating steps of fibrotic adhesion formation (She et al., 2026; Diamond and Freeman, 2001). Carboxymethyl chitosan (CMCS) exerts anti-adhesion effects after laparotomy through multiple mechanisms, including inhibition of fibrotic processes and formation of a surface water barrier reducing cell adhesion and migration (She et al., 2026).

Functional factor—Ceffe: Ceffe (cell-free fat extract) is obtained through mechanical homogenization, centrifugation, and filtration of adipose tissue and contains a composite bioactive mixture of growth factors, exosomes, and extracellular matrix proteins (She et al., 2026). Its therapeutic effects encompass multiple mechanisms of anti-inflammation and pro-repair, including attenuation of oxidative damage, enhancement of angiogenesis, and promotion of cell proliferation/migration (She et al., 2026).

#### 3.2.2 Key performance and biological effects

The BH patch demonstrated excellent mechanical strength, prolonged post-swelling adhesion retention, and enzyme resistance (She et al., 2026). Although the specific numerical values of lap shear adhesion strength were presented in the figures of the original publication rather than explicitly stated in the text, the investigators validated functional efficacy under simulated surgical conditions through wet-environment adhesion experiments and sealing tests. *In vitro* and *in vivo* studies confirmed favorable biocompatibility and anti-adhesion effects—including anti-adhesion performance evaluated via a cecal injury model and repair-promoting capacity assessed through tube-formation and scratch assays (She et al., 2026).

In a rat DP model, the Ceffe@BH group demonstrated significantly reduced POPF rates compared with the saline group and the manual suture group. The following data reflect its multi-dimensional therapeutic effects (She et al., 2026): reduced drain amylase indicating effective sealing of the pancreatic remnant; attenuated inflammation with decreased levels of TNF-α and IL-6; systematic analysis of eight inflammatory markers—VEGF, TNF-α, MIP-1α, GM-CSF, IL-1β, IL-6, GRO, and IL-17—with Ceffe@BH treatment not only reducing these markers but restoring them to normal levels; enhanced angiogenesis, with VEGF levels in the Ceffe@BH group slightly higher than in the postoperative normal group, possibly attributable to Ceffe-promoted proliferation of endothelial cells at the pancreatic resection margin; and effective adhesion prevention verified by H&E staining and immunofluorescence (She et al., 2026).

#### 3.2.3 Design advantages and evidence limitations

The most important design innovation of the BH patch lies in its “suture-free” strategy. The investigators explicitly recognized that conventional manual suturing in pancreatic surgery has “clear limitations”—the shear forces generated by excessive suture tension may tear fragile pancreatic tissue and trigger POPF (She et al., 2026)—and that replacing suturing methods can “reduce ischemic damage and inflammation risks” (She et al., 2026). The BH patch achieves tissue apposition through adhesion rather than suturing, circumventing suture-related injury in principle.

However, this study has significant limitations: (1) validation was performed only in a rat DP model—the microenvironment of DP (where pancreatic remnant leakage is predominantly in the form of zymogens, with protease activation depending on anatomical context) may differ substantially from that of PD (which involves enterokinase-triggered cascade activation), and the applicability of the BH patch design to PD procedures has not been evaluated; (2) no head-to-head *in vivo* comparison with other sealant materials was conducted; (3) Ceffe, as a biologically-derived extract, presents challenges of batch-to-batch consistency and quality control standardization of its growth factor and extracellular matrix components, which constitute core challenges for clinical translation (see Section 4.4); and (4) the feasibility of delivering a pre-formed bilayer patch through a trocar during laparoscopic minimally invasive surgery has not been explored (see Section 4.3).

#### 3.2.4 Comparison and synthesis: comparative analysis of adhesion strategies

The adhesion strategies of POPF hydrogels in the published literature can be grouped into three major categories:

1. Physicochemical synergistic adhesion (BH patch, She et al., 2026): NHS covalent bonding plus xanthan gum interfacial dehydration plus UV polymerization. Strengths include strong and durable adhesion with concurrent enzyme resistance. It should be noted that the specific lap shear adhesion strength values of this study were presented in the figures of the original publication and not explicitly stated in the text—this limits direct quantitative comparison with other systems. Limitations include the requirement for UV irradiation and a certain operative time window.
2. Photoactivated radical crosslinking adhesion (CGO@Pg-Cu(II), She et al., 2026): UVA light (365 nm, 7 mW/cm^2^, 10 s)-activated radicals form C-C covalent bonds with intrinsic tissue molecules, achieving “transient unilateral adhesion.” She et al. 2026 employed adhesion strength and storage modulus (G') as optimization parameters to determine the optimal formulation of 13.5% total solid content and CF-GMA:OP = 2:1, although the specific lap shear values are likewise presented in figures. The strengths of this strategy include spatial controllability (adhesion only to the pancreas-facing side) and rapid execution. Limitations include the requirement for UVA irradiation equipment, with the feasibility of uniform irradiation within the confined space of laparoscopic surgery remaining unevaluated.
3. Physical adsorption and elastic sealing (PVA sheet, Kemmochi et al., 2021; SPG-178, Mikamori et al., 2022): reliance on physical coverage and passive contact of the hydrogel with tissue. Strengths include simplicity of application. Limitations include weak adhesion and poor interfacial stability in a dynamic moist environment—this may partly explain why, although the PVA sheet in Kemmochi et al. (2021) was effective (ascites amylase 303.1 IU/L vs. control 4992.4 IU/L), its variability remained large (SD 240.1 IU/L), and why SPG-178 reduced ascites amylase but the effect size information was incompletely reported.

Trend assessment: The transition from passive physical adsorption to covalent/coordination bonding appears to be the clear trend in adhesion strategy for this field—both the BH patch and CGO@Pg-Cu(II) represent this direction. However, an inherent tension exists between stronger adhesion (generally implying more stable covalent bonds) and “on-demand degradation” (requiring bond reversibility or hydrolyzability). How to strike a balance between adhesion durability and timely degradation remains a core unresolved challenge in this field (see Section 4.2 for details). It must be candidly acknowledged that, due to the lack of a unified adhesion strength testing standard across studies (test conditions for lap shear, burst pressure, and sealing tests are mutually inconsistent), quantitative comparison of adhesion across systems is currently infeasible—establishing a standardized adhesion assessment protocol represents an urgent need in this field.

### 3.3 Strategy III: PVA-based platforms for antibacterial, anti-inflammatory, and pro-healing multifunctional integration

PVA is among the most widely used base polymers in the published POPF hydrogel literature. From the pure PVA freeze-thaw sheet of Kemmochi et al. (2021) to the PVA/QCS/AgNP dual-crosslinked antibacterial system of Zhou et al. (2026) and the PC-OHAD@MP multifunctional hydrogel of Zhou et al. (2025), the PVA platform has undergone a remarkable functional evolution—a trajectory that itself serves as an excellent case study for understanding the design logic of “multifunctional integration.”

#### 3.3.1 From pure physical sealing to multifunctionality: the evolutionary trajectory of the PVA platform

Starting point—pure physical barrier: The PVA hydrogel sheet was prepared by the freeze-thaw method by Kemmochi et al. (2021) without any functional additives. At 48 h postoperatively, the sheet “remained in place” in the setting of substantial pancreatic fluid leakage. Key data: ascites amylase 303.1 ± 240.1 IU/L (vs. control 4992.4 ± 5355.7 IU/L, P < 0.05); ascites lipase 86.8 ± 59.8 IU/L (vs. control 2279.8 ± 3395.2 IU/L, P<0.05). Comparison with commercial products: VIEWGEL group ascites amylase 1068.4 ± 269.1 IU/L, Neoveil group 730.2 ± 425.2 IU/L—the PVA sheet was numerically superior to both and the differences were statistically significant (P < 0.05) (Kemmochi et al., 2021). The contribution of this study lies in providing the first proof of feasibility for hydrogel-based PF prevention. However, the limitations are equally apparent: only physical sealing was achieved—no antibacterial function, no enzyme-resistance strategy, and no bioactivity—essentially remaining a “passive barrier.”

First functional upgrade—antibacterial plus pro-healing: The PVA/QCS/AgNP hydrogel of Zhou et al. (2026) achieved dual crosslinking through UV reduction of Ag^+^ and freeze-thaw cycling. Two key upgrades were introduced to the original PVA system: (1) incorporation of quaternized chitosan (QCS)—cationic charge disrupts bacterial membranes, providing intrinsic antibacterial activity; (2) *in situ* synthesis of silver nanoparticles (AgNPs)—sustained Ag^+^ release induces oxidative stress, achieving broad-spectrum antibacterial effects. The design maintained greater than 90% fibroblast viability, indicating that antibacterial functionality was not achieved at the expense of biocompatibility (Zhou et al., 2026).

This study features one of the most systematic degradation testing protocols in the published literature—a six-condition degradation matrix (pH 5.4/7.4/8.0 × PBS/SPF) that revealed the synergistic influence of multiple factors on degradation rate. Notably, the pH 5.4+SPF (acidic plus simulated pancreatic fluid) condition exhibited the fastest mass loss, indicating a synergistic accelerating effect between acid-catalyzed hydrolysis and enzymatic cleavage (Zhou et al., 2026). Importantly, even under the alkaline condition (pH 8.0) with SPF—the simulated condition closest to the authentic POPF microenvironment—the degradation rate of the hydrogel was substantially faster than in PBS at the same pH, confirming that enzyme activity is the primary driver of degradation. The release rate of Ag^+^ in SPF was faster than in PBS (particularly at early-to-mid time points, days 1–7), consistent with the mechanism that enzymatic degradation accelerates hydrogel network collapse, thereby promoting Ag release (Zhou et al., 2026).

In the rat PF model, the hydrogel reduced fluid leakage and inflammation, accelerated collagen deposition, and normalized serum amylase levels within 14 days (Zhou et al., 2026). Transcriptomic analysis revealed the underlying mechanism—upregulation of cell cycle progression and ECM remodeling pathways, coupled with suppression of pro-inflammatory cytokine expression—providing a molecular-level explanation for its “dual role in infection control and tissue regeneration” (Zhou et al., 2026).

Second functional upgrade—targeted antibacterial plus NF-κB anti-inflammatory plus degradation-healing matching: The PC-OHAD@MP hydrogel of Zhou et al. (2025) employs PVA, chitosan (CS), and dopamine-modified oxidized hyaluronic acid (OHAD) as a three-component backbone (mass ratio OHAD/CS/PVA = 1:1:2), loaded with the broad-spectrum antibiotic meropenem (MP, 0.17 mg/mL), and physically crosslinked through three freeze-thaw cycles (−20 °C/20 h, 25 °C/4 h) (Zhou et al., 2025).

The innovation of this system lies in simultaneously addressing both antibacterial spectrum breadth and anti-inflammatory mechanism: (1) meropenem covers the critical Gram-negative bacilli (*E. coli*, *P. aeruginosa*, *K. pneumoniae*) and some Gram-positive cocci (*S. aureus*) encountered in intra-abdominal infections—all four tested strains were effectively inhibited (Zhou et al., 2025); (2) macrophage inflammatory responses were suppressed through the NF-κB pathway—compared with the LPS-treated group, P65, p-P65, and p-IκBα levels in the PC-OHAD@MP group were significantly reduced, while IκBα levels were elevated (Zhou et al., 2025). The NF-κB pathway is a core regulatory node in the POPF-related inflammatory cascade; its inhibition implies the systematic blockade of multiple upstream pro-inflammatory mediators (TNF-α, IL-1β, IL-6) rather than targeting of a single cytokine.

Degradation-healing matching (see Section 3.3.2 for details) constitutes another core innovation of this system (Zhou et al., 2025).

#### 3.3.2 Quantitative matching of degradation kinetics with the anastomotic healing window

PC-OHAD@MP by Zhou et al. (2025) is the only study among those reviewed that explicitly correlates hydrogel degradation kinetics with specific anastomotic healing phases (Figure 3). In simulated pancreatic fluid (pH 8.0), the hydrogel retained approximately 60% of its mass after 14 days, “ensuring sustained mechanical support during the early healing phase (1–3 weeks), when mechanical integrity is critical”; it then continued to degrade beyond 20 days, “coinciding with the transition toward tissue remodeling (3–6 months), minimizing the risk of long-term foreign body reaction” (Zhou et al., 2025). The investigators explicitly correlated this degradation profile with “the healing cycle of pancreaticoduodenal anastomosis” [as cited in 25].

**FIGURE 3:**
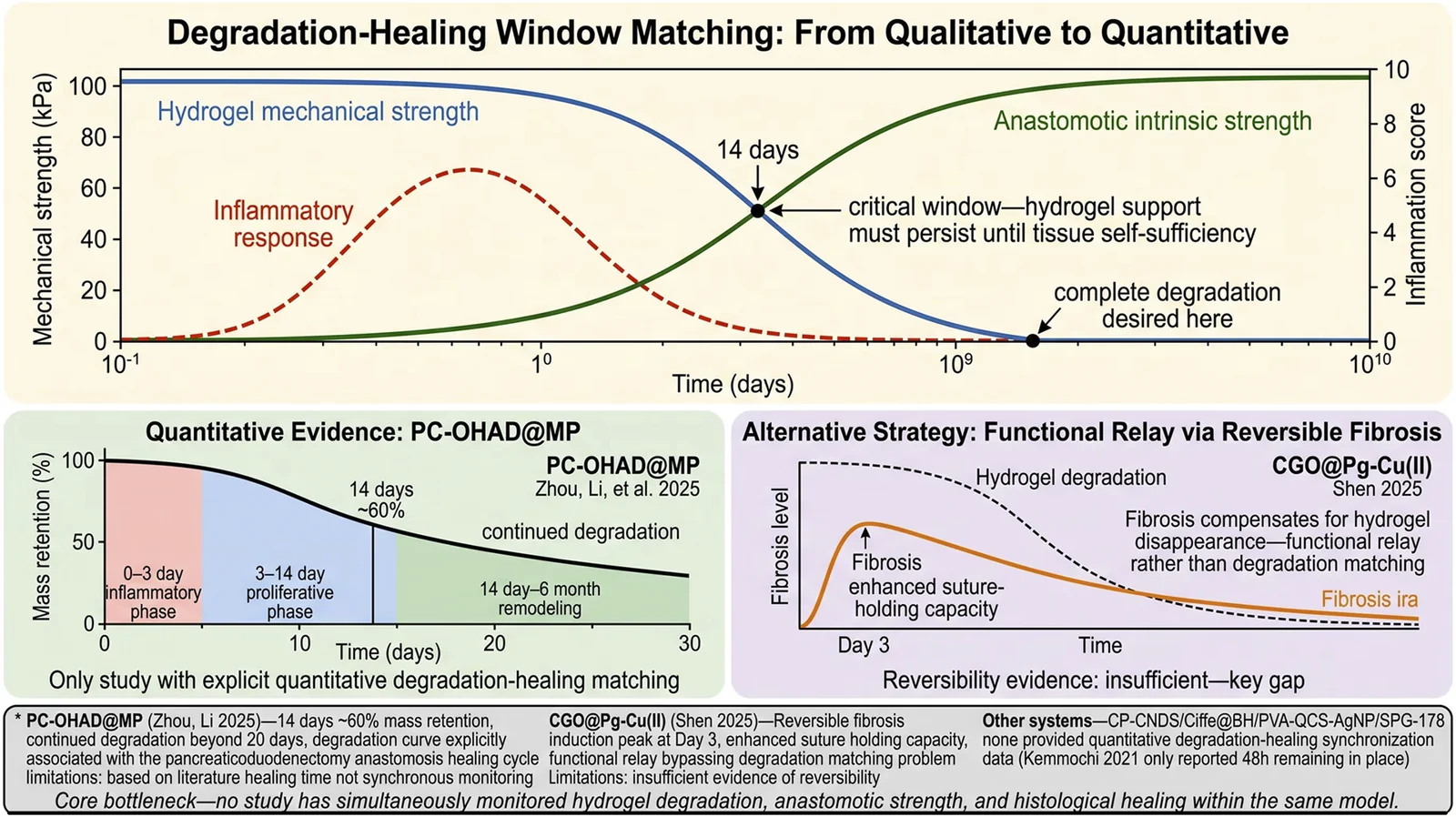
Degradation-healing window matching: theoretical framework and current evidence. Upper panel: This author-proposed conceptual framework illustrates the idealized relationship between hydrogel mechanical strength (decreasing sigmoid), anastomotic intrinsic strength (increasing sigmoid), and inflammatory response (bell-shaped curve) over postoperative time. The critical window lies between the intersection of the two strength curves—hydrogel support must persist until tissue self-sufficiency. Lower left: Quantitative evidence from PC-OHAD@MP (Zhou et al., 2025)—approximately 60% mass retention at 14 days with continued degradation beyond 20 days, correlated with pancreaticoduodenal anastomosis healing phases. Mass retention data indicate material persistence but do not directly demonstrate retained sealing function. Lower right: Alternative functional relay strategy from CGO@Pg-Cu(II) (Shen et al., 2025)—reversible fibrosis induction compensates for hydrogel disappearance, bypassing the degradation-matching challenge, though reversibility evidence remains insufficient. Footnote: The core bottleneck is that no study has simultaneously monitored hydrogel degradation, anastomotic mechanical strength, and histological healing within the same model.

It is important to distinguish between mass retention and retained sealing function. Mass retention data confirm that material remains present at a given time point, but do not directly demonstrate that the hydrogel continues to provide effective sealing, adhesion integrity, or burst-pressure resistance. The degradation process may compromise interfacial adhesion or cohesive integrity before substantial mass loss occurs. Therefore, the observed approximately 60% mass retention at 14 days provides a useful benchmark for material persistence, but cannot be interpreted as direct evidence of maintained sealing efficacy at that time point.

The clinical logic of temporal matching is sound: excessively rapid degradation—as with rapidly absorbable PGA mesh—would lead to premature loss of mechanical support, at a time when the anastomosis has not yet acquired sufficient intrinsic strength; excessively slow degradation or non-degradation—as with the long-term persistence of certain synthetic polymers—may provoke chronic inflammation and foreign body reaction. As the investigators noted: “short-term degradation can impede complete anastomotic closure, while long-term non-degradation can lead to tissue adhesion and biological toxicity” (Zhou et al., 2025).

However, this matching is based on typical healing timeline data reported in the literature, rather than on simultaneous monitoring of degradation and healing progression within the same model—this constitutes a key gap in the current evidence (see Section 4.2 for details).

#### 3.3.3 Systematic comparison of antibacterial mechanisms

PVA/QCS/AgNP (Zhou et al., 2026) and PC-OHAD@MP (Zhou et al., 2025) represent two fundamentally different antibacterial strategies. QCS plus AgNPs provides dual physical-chemical bactericidal action: QCS interacts electrostatically with the negatively charged bacterial cell membrane via its cationic charge, leading to increased membrane permeability and leakage of cellular contents; AgNPs release Ag^+^, inducing ROS generation and oxidative stress damage. The strengths of this strategy include broad-spectrum activity and reduced propensity for inducing resistance (multiple mechanisms), but the local and systemic cumulative toxicity of Ag^+^ and the fate of AgNPs following hydrogel degradation require long-term evaluation. Meropenem represents classical pharmacological antibacterial action through inhibition of bacterial cell wall synthesis (binding to penicillin-binding proteins). Strengths include a well-defined mechanism, extensive clinical experience, and a precisely characterized antibacterial spectrum. Limitations include risk of resistance development (particularly carbapenemase-producing strains), potential effects of high local concentrations on the normal microbiota, and the demands of precise control over antibiotic loading and release profiles.

A critical distinction is that AgNPs exhibit accelerated release in the microenvironment of enhanced enzymatic degradation (Zhou et al., 2026), forming an “activity-responsive” release; whereas meropenem release primarily follows passive diffusion, governed by hydrogel network density and swelling degree (Zhou et al., 2025). The advantage of activity-responsive release lies in the principle that more drug is released where it is most needed (higher enzyme activity, greater infection risk)—but it must be ensured that the released concentration remains within the therapeutic window rather than reaching toxic levels.

#### 3.3.4 Comparison and synthesis: evolutionary logic and unresolved questions of the PVA platform

The evolution of the PVA platform reveals a deep design logic: functional addition is not mere stacking but requires the fine-tuning of chemical-physical networks to achieve synergy and balance among different functions (Zhou et al., 2026; Kemmochi et al., 2021; Zhou et al., 2025). Specifically:

Balance between antibacterial activity and biocompatibility: The AgNP concentration design of Zhou et al. (2026) and the meropenem loading of Zhou et al. (2025) (0.17 mg/mL) were both screened through cell viability assays (greater than 90% and favorable biocompatibility, respectively), reflecting the introduction of the “therapeutic window” concept.

Coupling of degradation and function: The Ag^+^ release rate correlates with the hydrogel degradation rate (Zhou et al., 2026), meaning that the degradation curve not only determines the durability of mechanical support but also regulates the delivery kinetics of antimicrobial silver ions—degradation and function are intrinsically coupled. This may represent a double-edged sword: during the early treatment phase, high antibacterial activity and slow degradation are both desirable to ensure barrier integrity, yet accelerating degradation to achieve greater Ag release would weaken the barrier (Zhou et al., 2026).

Quantitative comparison of degradation kinetics: Among the two PVA-based systems, PC-OHAD@MP provides explicit mass retention data (14 days approximately 60% mass retention, pH 8.0 SPF) (Zhou et al., 2025), while PVA/QCS/AgNP’s six-condition matrix reveals the synergistic effects of pH and SPF on degradation rate (Zhou et al., 2026). Regrettably, the degradation testing conditions of the two are not directly comparable (single-condition pH 8.0 SPF for the former versus six-condition matrix with specific numerical values in figures for the latter), limiting direct quantitative comparison. Degradation data for the BH patch and CGO@Pg-Cu(II) were not reported in quantitative form in the text of the available literature—underscoring the need for the field to establish a unified, clearly citable degradation testing reporting standard.

Trade-offs between preparation process and function: The freeze-thaw method (physical crosslinking) avoids the introduction of chemical crosslinkers, offering superior biocompatibility, but the controllability and reproducibility of network structure are inferior to those of chemical crosslinking. UV crosslinking (Zhou et al., 2026) or three-component dynamic covalent crosslinking with OHAD (Zhou et al., 2025) provides more precise network control but introduces potential biocompatibility considerations associated with photoinitiators or chemical reagents.

Unresolved questions: (1) Head-to-head comparisons among the three PVA hydrogels are lacking—which system performs best in the rat PF model? Does functional addition indeed translate into superior *in vivo* efficacy? (2) The specific chemical mechanisms of PVA hydrogel degradation in pancreatic fluid—whether oxidative cleavage of the PVA backbone, enzymatic hydrolysis of CS, or dissociation of dynamic bonds in OHAD predominates—have not been elucidated. (3) Long-term biodistribution and organ accumulation data for AgNPs or meropenem in the peritoneal cavity are absent.

### 3.4 Strategy IV: temporal regulation, immunomodulation, and full-cycle management

The pathological process of POPF is not a static event but rather a dynamic cascade unfolding along a temporal axis: immediate postoperative leakage and hemorrhage (minutes to hours) → acute inflammation and infection (24–72 h) → initiation of tissue repair (3–7 days) → remodeling and healing (weeks to months). Recognizing this temporal dimension, two studies have proposed integrative therapeutic frameworks that transcend single-function approaches (Shen et al., 2025; Pausch et al., 2022).

#### 3.4.1 Temporal design of “full-cycle Management”—CGO@Pg-Cu(II)

The “full-cycle PF management” framework proposed by Shen et al. (2025) represents a systematically conceived and temporally refined therapeutic framework in the published literature. According to their protocol (Shen et al., 2025), this strategy partitions the PF healing process into four temporally-defined intervention phases:

Phase 1—Immediate postoperative period (Day 1)—rapid hemostasis and sealing: The hydrogel adapts to irregular PF wounds through injectable self-leveling and, upon UVA light irradiation (365 nm, 7 mW/cm^2^, 10 s, 0.15 wt% I2959 photoinitiator), achieves radical crosslinking with the formation of covalent bonds to intrinsic tissue molecules—“transient unilateral adhesion” (Shen et al., 2025). This ensures that the hydrogel adheres only to the pancreas-facing side, avoiding non-specific adhesion to surrounding tissues. The use of UVA irradiation raises considerations regarding residual photoinitiator cytotoxicity, potential photochemical or thermal injury to the pancreas and adjacent intestine and vasculature, and the impact of blood and tissue occlusion on cure depth—factors beyond the “rapid crosslinking in 10 s” emphasized in the original study.

Phase 2—Intervention window (Days 2–5)—local anti-inflammation: CGO@Pg-Cu(II) sustains the release of Pg-Cu(II) ICPs along a concentration gradient via passive diffusion (maximum concentration not exceeding 350 μg/mL to ensure biocompatibility) (Shen et al., 2025), suppressing excessive immune activation and reducing infection risk.

Phase 3—Peak phase (approximately Day 3)—reversible fibrosis induction: This is an original and potentially debated dimension of the strategy. Conventionally, postoperative fibrosis has been regarded as a complication to be avoided. However, Shen et al. (2025), proceeding from surgical needs, proposed the utilization of penicillin G to induce controlled, reversible local fibrosis—mechanically reinforcing the pancreatic remnant by enhancing suture-holding capacity and reducing pancreatic exocrine secretion, thereby lowering the risk of secondary PF. The potential long-term consequences of this approach warrant careful consideration: effects on pancreatic exocrine and endocrine function (including diabetes risk in the setting of pre-existing chronic pancreatitis (Malka et al., 2000)), the risk of anastomotic stricture, the possibility of chronic pancreatitis-like changes, and the additional safety burden of proof this strategy would face during regulatory review for a benign surgical indication.

The molecular engineering challenge in realizing this objective lies in the nature of penicillin G: a β-lactam antibiotic with extremely high water solubility (330 mg/mL), an exceedingly short *in vivo* half-life (30–60 min), and an inability to passively diffuse across the lipid bilayer of mammalian cells due to the absence of hydrophobic moieties. Shen et al. (2025) addressed this through Cu(II) coordination nanoengineering—employing the phenolic hydroxyl, carboxyl, and carbonyl groups of penicillin G as multidentate ligands to form infinite coordination polymers of approximately 7.11 nm with hexacoordinate Cu(II) ions—successfully achieving: (1) nanoengineering to overcome the cell uptake barrier; (2) pH-responsive lysosomal release (coordination polymers dissociate at acidic pH 4.5–6.3); and (3) intrinsic trypsin-inhibitory activity of the Cu(II) ions themselves (Shen et al., 2025).

Phase 4—Remodeling phase (greater than Day 5)—promotion of tissue repair: The hydrogel promotes regenerative repair of pancreatic remnant tissue (Shen et al., 2025).

Core efficacy data: CGO@Pg-Cu(II) hydrogel achieved 100% anti-PF efficacy at Day 7 (Shen et al., 2025).

Evidence level and limitations: This study provides a detailed theoretical framework and impressive efficacy data, but the depth of validation is uneven across phases—evidence for the “reversibility” of fibrosis induction (whether fibrosis completely resolves on long-term follow-up, and its impact on pancreatic exocrine function) and long-term safety data are currently insufficient. The investigators themselves acknowledged that “further optimization and validation in large-animal studies are essential before clinical translation” (Shen et al., 2025).

#### 3.4.2 Dual-functional integration of POPF prevention and tumor immunotherapy

The (P-aTIGIT&PDA@IL-15)@gel hydrogel reported by Zhao et al. in Biomaterials in 2024 (Pausch et al., 2022) represents another unique dimension of integration—simultaneously addressing POPF and postoperative pancreatic cancer recurrence. For patients undergoing surgical resection for pancreatic ductal adenocarcinoma (PDAC), the postoperative period presents two major threats: POPF and tumor recurrence (from residual microdeposits). No prior unified strategy had been able to simultaneously address both.

Design rationale: The hydrogel, based on dual-crosslinked hyaluronic acid (MA-HA-DA and SH-HA-DA), constructs an immunostimulatory drug depot loaded with two immunotherapeutic agents: (1) polydopamine-encapsulated interleukin-15 (PDA@IL-15)—IL-15 is a critical activator of CD8^+^ T cells and NK cells; and (2) platelet-conjugated anti-TIGIT antibody—TIGIT is an immune checkpoint molecule on T cells, and blockade of TIGIT can relieve immunosuppression within the tumor microenvironment (Pausch et al., 2022). Conceptually, the integration of local drug delivery with multimodal tumor treatment in an implantable format has been explored in adjacent oncology fields; for instance, pH/NIR dual-responsive nanofiber patches combining photothermal therapy with chemotherapy have demonstrated the feasibility of spatially and temporally controlled combination therapy for solid tumors (Sun et al., 2026). While such systems were not designed for pancreatic surgery, their conceptual framework—localized delivery achieving both physical treatment and drug-based intervention—shares methodological parallels with dual-functional POPF/tumor-recurrence prevention.

The incorporation of PDA nanoparticles and platelets serves a dual function: beyond their role as carriers for immunotherapeutic agents, they also “protect the immunotherapeutic agents from degradation by proteases in pancreatic fluid” (Pausch et al., 2022)—resolving the stability challenge of bioactive factors in the pancreatic microenvironment.

Dual-model validation: In a rat PF model, the hydrogel effectively adhered to the pancreatic remnant, sealed the stump, and prevented postoperative elevations of amylase and lipase. In a pancreatic cancer mouse model, the hydrogel, through sustained release of IL-15 and anti-TIGIT antibody, effectively activated CD8^+^ T cells and NK cells, suppressing residual tumor growth and distant metastasis—the bioluminescent signal of liver metastases was significantly reduced (P<0.01) (Pausch et al., 2022). However, it should be noted that these two functional dimensions were validated in two separate animal models—PF prevention and tumor recurrence suppression were not simultaneously evaluated in a single model combining pancreatic cancer resection with PF risk (Pausch et al., 2022).

Evidence level: Proof-of-concept study. Limitations: the two functional dimensions were not simultaneously validated in a single model; the investigators explicitly called for “evaluation of the efficacy and safety of this therapeutic strategy in large animal models to pave the way for clinical translation” (Pausch et al., 2022).

#### 3.4.3 Comparison and synthesis: systematic analysis of temporal design and immunomodulation

Juxtaposing the full-cycle management of CGO@Pg-Cu(II) (Shen et al., 2025), the dual-functional integration of Zhao et al. (Pausch et al., 2022), and the restoration of inflammatory markers to normal levels by Ceffe@BH (She et al., 2026) along the immunomodulatory dimension reveals the following patterns:

Hierarchy of intervention depth: The immunomodulation of Ceffe@BH is the least deep yet safest—multi-target modulation of the inflammatory microenvironment through a natural mixture of bioactive factors (growth factors, exosomes), without involving interventions at the level of specific gene expression (She et al., 2026). However, it is important to distinguish beneficial suppression of excessive inflammation from excessive immune inhibition that may impair the controlled inflammatory response required during early tissue repair. “Normalization” of inflammatory markers does not necessarily indicate an optimal immune state for wound healing; a moderately elevated inflammatory milieu may be physiologically appropriate in the early postoperative phase. The immunomodulation of Zhao et al. (Pausch et al., 2022) is the deepest—specific activation of CD8^+^ T and NK cells via IL-15 and anti-TIGIT antibody—but also carries the highest risk, including potential for excessive immune activation, inflammatory amplification, or immune-related adverse effects. The temporal balance among inflammation control, tissue repair, and antitumor immunity represents an under-explored dimension that warrants systematic investigation. CGO@Pg-Cu(II) occupies an intermediate position—exerting broad-spectrum anti-inflammatory effects through NF-κB pathway inhibition, while simultaneously achieving the unique synergy of “immunomodulation promoting structural reinforcement” through reversible fibrosis (Shen et al., 2025).

Maturity of temporal design: CGO@Pg-Cu(II) is currently the only therapeutic framework with clearly delineated temporal phases (Shen et al., 2025). Although the studies by Zhao et al. (Pausch et al., 2022) and (She et al., 2026) incorporate a temporal dimension (sustained release), they do not set “which function to achieve at which time point” as an explicit design objective. The methodological value of the full-cycle management concept for the field lies in its elevation of hydrogel design from a “spatial problem” (sealing a leak point) to a “spatiotemporal problem” (sequentially executing multiple functions along the healing timeline).

Differences in therapeutic goals: She et al. (2026) and Shen et al. (2025) address PF prevention in a benign scenario—only the healing of the remnant is required. Zhao et al. (Pausch et al., 2022) address a malignant scenario—not only must the remnant heal, but tumor recurrence must also be simultaneously suppressed. This distinction imposes fundamentally different requirements on hydrogel degradation: the former requires degradation to coordinate with the normal healing process (3–6 months), while the latter may require more prolonged sustained immunostimulatory release to cover the high-risk window for tumor recurrence (typically 1–2 years postoperatively).

## 4 Key gaps in clinical translation

Despite the encouraging preclinical efficacy demonstrated by the aforementioned multifunctional hydrogels at the proof-of-concept level, one fact must be candidly confronted: all reviewed multifunctional hydrogel studies remain at the preclinical stage, and no therapeutic multifunctional hydrogel product has yet entered clinical trials. Two historical notes provide context: an early single-function PEG-based photopolymerizable hydrogel sealant (FocalSeal-L) was evaluated in a canine DP model, demonstrating reduced pancreatic drainage (25 ± 5 mL vs. 91 ± 26 mL, P = 0.05 at Day 6) with intact sealant at 14 days post-implantation (Balcom et al., 2002), but this technology did not progress to clinical use and lacked the multifunctional features (enzyme resistance, antibacterial activity, immunomodulation) that define the current generation of materials under review. Separately, non-hydrogel synthetic sealants and patches—including PEG/recombinant human albumin sealants (Privett et al., 2021), PEG-coated collagen patches (Serradilla-Martín et al., 2023), and biodegradable polyurethane bilayer patches (Goetz et al., 2024)—have entered clinical evaluation for POPF prevention, representing parallel but distinct translational paths outside the multifunctional hydrogel scope. This chapter analyzes the translational gaps from five dimensions, based on evidence specifically reported or limitations explicitly identified in the published literature (Figure 4).

**FIGURE 4:**
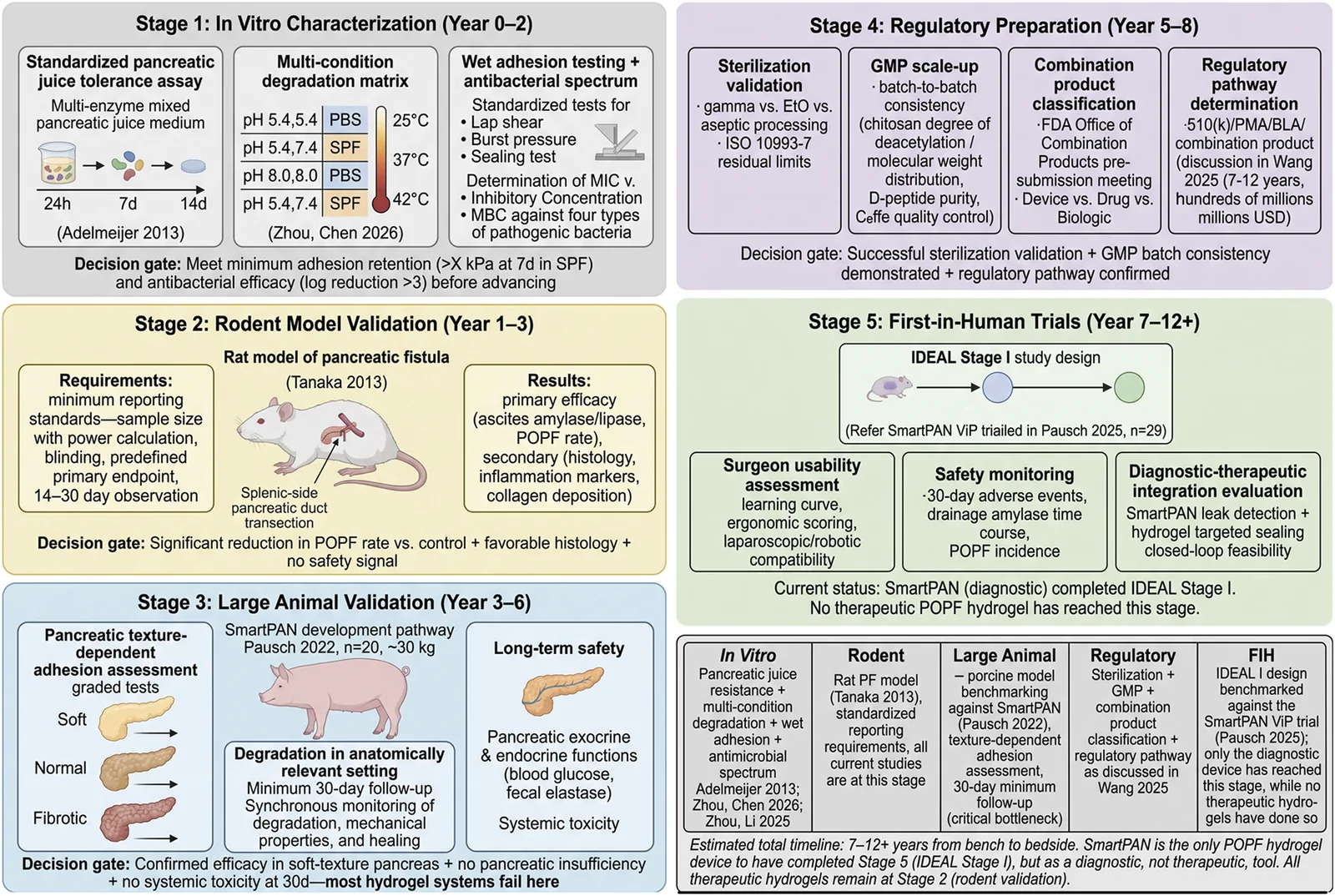
Clinical translation pathway for POPF hydrogels: from bench to bedside. This author-proposed conceptual framework should be interpreted as a suggested roadmap rather than an empirically validated pathway. Stages 1–3 (left panel): Stage 1—In vitro characterization including standardized pancreatic juice tolerance assays (Adelmeijer et al., 2013), multi-condition degradation matrices (Zhou et al., 2026), and wet adhesion testing. Stage 2—Rodent model validation using the rat PF model (Tanaka et al., 2013) with minimum reporting standards (power calculation, blinding, 14–30 days observation). All reviewed therapeutic multifunctional hydrogels remain at this stage. Stage 3—Large animal validation in a porcine model, requiring texture-dependent adhesion assessment, simultaneous degradation-mechanics-healing monitoring, and pancreatic function safety evaluation. An early single-function PEG hydrogel sealant was previously evaluated in a canine DP model (Balcom et al., 2002) but did not progress to clinical use. Separately, non-hydrogel synthetic sealants and patches have entered clinical evaluation through parallel translational paths (Privett et al., 2021; Serradilla-Martín et al., 2023; Goetz et al., 2024). Among reviewed multifunctional hydrogels, none has completed this stage. Stages 4–5 (right panel): Stage 4—Regulatory preparation encompassing sterilization validation per applicable ISO standards (effects are highly formulation-dependent), GMP scale-up, and combination product classification (Wang et al., 2025). Stage 5—First-in-human trials following the IDEAL Stage I design modeled by the SmartPAN ViP trial (Pausch et al., 2025) (n = 29, single-arm, preliminary safety and usability data). SmartPAN is the only POPF-related hydrogel device to have entered human trials, as a diagnostic leak indicator rather than a therapeutic sealant. Footnote: Estimated total timeline 7–12+ years. All reviewed therapeutic multifunctional hydrogels remain at Stage 2.

### 4.1 Species disparities in animal models and predictive validity

Evidence landscape: The *in vivo* validation of POPF hydrogels in the published literature is almost entirely based on rat models—including CP-CNDS (He et al., 2024), PVA/QCS/AgNP (Zhou et al., 2026), PVA sheet (Kemmochi et al., 2021), SPG-178 (Mikamori et al., 2022), CGO@Pg-Cu(II) (Shen et al., 2025), (P-aTIGIT&PDA@IL-15)@gel (Pausch et al., 2022), PC-OHAD@MP (Zhou et al., 2025), and Ceffe@BH (She et al., 2026). The sole large-animal exception among reviewed hydrogel studies is SmartPAN—Pausch et al. (Yamashita et al., 2013) validated its efficacy in a porcine model (n = 20, German domestic pigs, approximately 30 kg) and employed human pancreatic juice for *ex vivo* testing. The development pathway of SmartPAN—adherence to the IDEAL framework, screening of 41 prototypes, porcine model validation prior to human trial entry—provides a replicable methodological paradigm for the translation of therapeutic POPF hydrogels (Pausch et al., 2025; Yamashita et al., 2013).

It warrants specific emphasis that the currently published rat PF model studies exhibit a general deficiency in methodological transparency—the sample sizes (number of animals per group) of most studies were not explicitly reported in the accessible literature text (see Table 3 for details). The absence of sample size information not only limits independent assessment of evidence quality but also impedes the estimation of confidence intervals for effect sizes. This represents a fundamental issue requiring improvement in the methodological reporting standards of this field.

**TABLE 3:** Comparison of In vivo efficacy and safety of multifunctional POPF hydrogels.

Hydrogel system	Model	PF induction method	Sample size	Observation period	Primary efficacy endpoint	Key efficacy data	Secondary endpoints	Safety	Reference
PVA sheet	Rat	Pancreatic duct + parenchymal transection	Not reported	48 h	Ascites amylase/lipase	Amylase 303.1 vs. 4992.4 IU/L (P < 0.05); Lipase 86.8 vs. 2279.8 IU/L (P < 0.05)	Comparison with Neoveil/VIEWGEL	Remained in place at 48 h; systemic toxicity not reported	Kemmochi et al. (2021)
SPG-178	Rat	Splenic-side duct transection	Not reported	3 days	Ascites amylase	SPG-178 group significantly lower than untreated	Pathology: inflammatory cells decreased; fibrous tissue/mesothelium formation	Long-term toxicity not reported	Mikamori et al. (2022)
CP-CNDS	Rat	POPF model	Not reported	3–5 days	Survival	3-day recovery; mock group <5-day survival	Complete enzyme activity suppression	Long-term safety not assessed	He et al. (2024)
(P-aTIGIT&PDA@IL-15)@gel	Rat (PF); Mouse (PC)	PF model; PC subcutaneous + liver metastasis	Not reported	Not reported	PF: amylase/lipase; PC: liver metastasis signal	PF: prevented amylase/lipase elevation; PC: liver metastasis signal decreased (P < 0.01)	CD8^+^ T/NK cell activation	Not reported; calls for large-animal validation	Zhao et al. (2024)
CGO@Pg-Cu(II)	Rat	PF model	Not reported	7 days	Day 7 anti-PF efficacy	100%	Hemostasis, anti-inflammation, reversible fibrosis, tissue repair	Long-term safety not reported (fibrosis reversibility to be verified)	Shen et al. (2025)
Ceffe@BH	Rat DP	DP model	Not reported	Not reported	POPF rate; drain amylase	Ceffe@BH significantly decreased POPF vs. saline/manual suture	8 inflammatory markers restored to normal; VEGF increased; anti-adhesion	Favorable biocompatibility; cecal injury model validation	She et al. (2026)
PC-OHAD@MP	Rat	PF model	Not reported	Not reported	Anastomotic sealing; pro-healing	Effective sealing; NF-κB pathway decreased (decreased P65/p-P65/p-IκBα)	Antibacterial (4 strains), cell proliferation/migration increased	Favorable biocompatibility; degradation-healing matching	Zhou et al. (2025)
PVA/QCS/AgNP	Rat	Pancreatectomy model	Not reported	14 days	Serum amylase	Normalized at 14 days	Fluid leakage decreased; inflammation decreased; collagen deposition increased; transcriptomics: cell cycle + ECM remodeling increased	>90% fibroblast viability	Zhou et al. (2026)
SmartPAN	Porcine (n = 20); Human (n = 29, IDEAL I)	DP (porcine); Partial pancreatectomy (human)	20 (porcine); 29 (human)	48 h (porcine); 30 days (human)	Leak detection sensitivity and specificity; clinical usability	Porcine: sensitive and specific; Human: 10/29 positive detection, 7 POPF cases	Human: no AEs; 16/16 surgeons rated easy to use	No safety signal (human); BTB non-toxic concentration (preclinical)	Pausch et al. (2025), Pausch et al. (2022)

Caution—Direct quantitative comparisons of efficacy across studies are precluded by substantial heterogeneity in animal models, PF, induction methods, observation periods, endpoint definitions, and assessment criteria. Sample sizes denoted as “Not reported in accessible text” reflect limitations of the available literature extracts rather than definitive absence from the original publications; readers are encouraged to consult the complete primary articles and supplementary materials.

Specific dimensions of species differences: (1) Pancreatic anatomy: The rat pancreatic duct system comprises four small ducts—gastric, duodenal, common, and splenic ducts—fundamentally differing from the human anatomy of a single main pancreatic duct draining into the duodenum (Elayat et al., 1995). The rat PF model established by Tanaka et al. (2013) involves selective transection of the splenic-side pancreatic duct—this simulates partial ductal injury, rather than the clinical scenarios of complete pancreatic remnant leakage in DP or pancreaticoenteric anastomotic leakage in PD. (2) Pancreatic texture: The rat pancreas is considerably more resilient than its human counterpart. Shen et al. (2025) described the human pancreas as “soft as tender tofu” with “extremely poor suture-holding capacity,” whereas the rat pancreas possesses better suture tolerance during surgical manipulation. This implies that adhesion strategies that perform adequately in rat models—including moderate physical adsorption—may completely fail on soft, fragile human pancreas tissue: the “minimum effective threshold” for adhesion strength may differ by orders of magnitude between species, yet this threshold has never been systematically determined. (3) Pancreatic enzyme concentration and activity: Rats and humans differ in pancreatic enzyme composition (trypsin isoform distribution, lipase activity levels) and basal secretion rates (Elayat et al., 1995). More importantly, pancreatic fluid at the pancreaticoenteric anastomosis in human PD is exposed to enterokinase secreted by the jejunal mucosal brush border—a component entirely absent from the simple pancreatic duct transection model in rats. In the rat model, pancreatic proteases exist predominantly as zymogens, though it should be noted that lipase and α-amylase are secreted in their active forms and do not require enterokinase-mediated activation. The absence of enterokinase-mediated trypsinogen activation means that the predominant proteolytic activity in the rat model may be systematically lower than that in human PD. (4) Immune system differences: Multiple differences exist between rat and human innate and adaptive immune systems, which may affect hydrogel-induced inflammatory responses, foreign body reactions, and fibrotic processes—with particular relevance to the long-term safety assessment of hydrogels involving immunomodulatory strategies (Shen et al., 2025; She et al., 2026; Pausch et al., 2022).

Unanimous calls from the published literature: Shen et al. (2025) stated that “further optimization and validation in large-animal studies are essential before clinical translation”; Zhao et al. (Pausch et al., 2022) similarly emphasized the need to “evaluate the efficacy and safety of this therapeutic strategy in large animal models”; Zhou et al. (2025) declared that “further optimization and validation in large animal studies are necessary before clinical translation.” These highly consistent calls from different research groups constitute a powerful consensus driving the field toward large-animal validation.

Hypothesis (to be validated): We hypothesize that systematic comparison of the adhesion strength, sealing efficacy, and degradation behavior of different hydrogel systems in a porcine model will reveal “interspecies effect size attenuation” unobservable in rat models—i.e., significant efficacy differences observed in rats may diminish or vanish in pigs—with direct implications for the design of human trials and the estimation of target effect sizes.

### 4.2 Precision matching of the degradation-healing window: from qualitative to quantitative

Uniqueness of existing evidence: PC-OHAD@MP by Zhou et al. (2025) is currently the only study among those reviewed that explicitly correlates the temporal profile of hydrogel mass retention with anastomotic healing phases—14 days approximately 60% mass retention corresponding to the early healing phase (1–3 weeks), with continued degradation beyond 20 days corresponding to the tissue remodeling phase (3–6 months). All other hydrogel studies—including CP-CNDS, CGO@Pg-Cu(II), Ceffe@BH, PVA/QCS/AgNP, SPG-178, and PVA sheet—lack quantitative degradation-healing synchronization data. Kemmochi et al. (2021) only reported that the PVA sheet “remained in place” at 48 h postoperatively, with its degradation status at later observation time points unknown. As noted in Section 3.3.2, mass retention data should not be equated with retained sealing function; the mass remaining at 14 days may not correspond to equivalent adhesive or cohesive integrity.

Core contradiction: An ideal degradation profile should satisfy the following: sustained mechanical support and sealing provided by the hydrogel until the anastomosis acquires sufficient intrinsic mechanical strength; once self-sustaining healing is achieved, the hydrogel should undergo rapid degradation to avoid long-term foreign body reaction and chronic inflammation. However, the precise timing of these two nodes—“the threshold time point at which the anastomosis acquires self-sustaining strength” and “the time point at which foreign body reactions begin to increase significantly”—lacks precise experimental data for human pancreatic surgery.

References from intersecting domains: Wang et al. (2025), in their review of pancreatic cancer hydrogel delivery systems, noted that “non-degradable or slowly degradable base materials may persist *in vivo* for extended periods, potentially triggering chronic inflammation or fibrotic reactions”; and that “even for biodegradable hydrogels, degradation kinetics are often influenced by the complex *in vivo* environment and are difficult to precisely control—conventional hydrolytic or enzymatic degradation methods suffer from unpredictable degradation rates and poor spatiotemporal controllability.” “Achieving matching between hydrogel degradation rates and therapeutic requirements still requires in-depth investigation, which is crucial for long-term implantation safety and efficacy” (Wang et al., 2025). Although the original context of this discussion is pancreatic cancer therapeutic hydrogels, its logical applicability to the POPF domain is consistent.

The alternative approach of Shen et al. (2025): Through the strategy of reversible fibrosis induction, “suture-holding capacity enhancement” and “fibrosis resolution” are temporally separated—relying not on the disappearance of the material itself, but on the modulation of the tissue response to achieve “functional relay.” The rationale of this approach is that it circumvents the technical difficulty of precise degradation-healing matching—as long as the fibrosis is truly reversible, the tissue can return to a functionally normal state even if the hydrogel is not completely degraded. However, the current evidence base for “reversibility” is insufficient (Shen et al., 2025).

Core bottleneck to be resolved: No study to date has simultaneously monitored the temporal changes of the following three dimensions within the same model: (1) changes in hydrogel mass/volume/mechanical strength; (2) mechanical strength of the anastomosis/remnant (burst pressure or tensile strength); and (3) histological healing markers (collagen deposition, epithelialization, resolution of inflammation). The absence of this simultaneous monitoring system constitutes the most fundamental methodological bottleneck for evaluating degradation-healing matching—without such a system, any claim of “matching” remains an indirect inference rather than a direct verification.

Recommended reporting standards: To address the heterogeneity in current testing methodologies, we suggest that future studies in this field adopt: (a) lap shear adhesion testing with reference to ASTM F2255, conducted on pancreatic tissue explants under controlled hydration conditions; (b) burst pressure testing with reference to ASTM F2392; (c) degradation studies conducted in multi-enzyme simulated pancreatic fluid (rather than single purified trypsin) at physiologically relevant pH (8.0–8.3) and temperature (37 °C); and (d) mandatory reporting of sample sizes with confidence intervals for effect sizes.

### 4.3 Surgical handlability and procedural adaptability

Fragmentary evidence in the published literature: Kemmochi et al. (2021) mentioned that “there is a report regarding the use of hydrogel sealant to prevent pancreatic fistula, but the sealant is difficult to use” [as cited in 18], suggesting that handlability was once an impediment to the adoption of early hydrogel sealants.

Recent studies have consciously responded to this issue: CGO@Pg-Cu(II) emphasizes its “injectable self-leveling” property—after mixing the backbone components, it can be directly injected onto irregular PF wound surfaces, uniformly covering them through self-leveling, followed by 10 s of UVA light irradiation to achieve crosslinking and unilateral adhesion (Shen et al., 2025). The BH patch adopts a “suture-free” design—achieving sealing through adhesion rather than suturing, simplifying the surgical procedure (She et al., 2026). PC-OHAD@MP can be directly applied to the pancreaticoenteric anastomotic surface, with “good conformity to the anastomotic site” and the capacity to “fill potential suture gaps” explicitly listed as design objectives (Zhou et al., 2025).

The neglected core dimension—adaptability to minimally invasive surgery: No study among those reviewed has evaluated the feasibility of hydrogel delivery during laparoscopic (LPD) or robotic (RPD) pancreatic surgery (Kendrick and Cusati, 2010). This gap is particularly conspicuous against the backdrop that leading pancreatic centers worldwide have comprehensively adopted minimally invasive approaches (Giulianotti et al., 2010). Specific, unexplored technical questions include: (1) Delivery of pre-formed patches: The BH patch, as a pre-formed bilayer patch, has not been validated for the integrity of unfolding, precision of application, or stability under pneumoperitoneum pressure after being rolled for delivery through a trocar of 12 mm or smaller. (2) Precision positioning of injectable hydrogels: The injectability of CGO@Pg-Cu(II) and PC-OHAD@MP has been demonstrated in open surgery, but within the constrained laparoscopic field of view (typically a 30-degree scope, limited operative triangulation), the precise injection of hydrogel onto specific locations of the anastomosis (particularly the posterior wall and superior margin of the pancreaticoenteric anastomosis) poses significant spatial operative challenges. Methods for confirming completeness of coverage under laparoscopic visualization have likewise not been established. (3) Laparoscopic feasibility of photocrosslinking: The UVA photocrosslinking step of CGO@Pg-Cu(II) (365 nm, 7 mW/cm^2^, 10 s) and the BH patch require irradiation. In the laparoscopic setting, the uniformity of light delivery via optical fiber to the deep surgical field, the effect of irradiation angle on crosslinking efficacy, the risk of non-target photodamage to surrounding normal tissues (bowel, blood vessels), the potential cytotoxicity of residual I2959 photoinitiator, and the attenuation of UVA by blood and tissue occlusion remain entirely unknown. (4) Operative time constraints imposed by gelation time: The pre-gel of CGO@Pg-Cu(II) forms an injectable, shape-adaptive semi-solid through dynamic imine bonds, with the final network structure subsequently locked in by UVA photocrosslinking (Shen et al., 2025). The viscosity change of the pre-gel prior to injection (gradual thickening over time) imposes a temporal window constraint on the surgical manipulation—the surgeon must complete positioning and light irradiation before the pre-gel loses injectability. The specific duration of this temporal window and its operability within the surgical workflow urgently require quantitative assessment.

Hypothesis (to be validated): A sprayable *in situ* gelation system—e.g., atomizing the hydrogel precursor solution via a pneumatically or CO_2_-driven dual-barrel syringe with simultaneous crosslinking onto the tissue surface—may be more suitable for minimally invasive pancreatic surgical scenarios than pre-formed patches or simple injection, as it could achieve 360-degree gapless coverage of irregular anastomoses. However, dispersion control and crosslinking uniformity of aerosolized spray under pneumoperitoneum conditions present additional technical challenges.

### 4.4 Scalable manufacturing, sterilization, and regulatory pathways

Attribution of evidence sources: The following discussion is partly based on the review by Wang et al. (2025) published in Materials Today Bio on hydrogel delivery systems for pancreatic cancer therapy. This review systematically discusses translational challenges including GMP manufacturing, quality control, batch-to-batch consistency, sterilization validation, and combination product regulatory classification, with some of its insights possessing cross-disease-domain applicability. However, it must be explicitly noted that the original context of this review is pancreatic cancer therapeutic hydrogels—hydrogels loaded with chemotherapeutic/immunotherapeutic agents differ from POPF-preventive hydrogels in regulatory classification, dosing frequency, and safety requirements. The following analysis indicates which discussions are direct inferences and which are extrapolations requiring independent validation in the POPF context.

Universal challenges in GMP scalable manufacturing (based on Wang et al. (2025)): Natural polymer hydrogels—such as chitosan (PC-OHAD@MP (Zhou et al., 2025), PVA/QCS/AgNP (Zhou et al., 2026)), hyaluronic acid (Zhao et al. (Pausch et al., 2022)), and recombinant collagen (CGO@Pg-Cu(II) (Shen et al., 2025))—despite favorable biocompatibility, “often face challenges of batch-to-batch variability and poor reproducibility, posing difficulties for large-scale production” (Wang et al., 2025). Specific challenges include: batch-to-batch variation in the degree of deacetylation and molecular weight distribution of chitosan directly affects the crosslinking density, mechanical properties, and degradation rate of hydrogels; the degree of modification of hyaluronic acid (e.g., dopamine grafting ratio and degree of oxidation in OHAD) affects adhesion strength and network stability. The batch-to-batch purity and sequence fidelity of synthetic peptides (D-amino acid peptide CP-CNDS (He et al., 2024), SPG-178 (Mikamori et al., 2022)) depend on the scalability of solid-phase synthesis and purification processes (Wang et al., 2025).

Specific sterilization challenges (inferential analysis, requiring POPF-specific validation for each final product formulation): The effects of sterilization on hydrogel properties are highly formulation-dependent and cannot be reliably inferred from the polymer name alone. Gamma irradiation may induce chain scission, crosslinking, or both in PVA-based hydrogels, depending on the radiation dose, hydration state, oxygen exposure, and the presence of other components; AgNPs may undergo agglomeration or oxidation. Autoclaving may disrupt physically crosslinked PVA microcrystalline domains, but the extent of this effect depends on the specific freeze-thaw protocol, water content, and the presence of stabilizing additives—it is not universally and irreversibly destructive. For hydrogels loaded with meropenem (PC-OHAD@MP (Zhou et al., 2025)) or Ceffe (BH patch (She et al., 2026)), elevated temperatures during autoclaving will cause drug or bioactive factor inactivation. Ethylene oxide (EtO): residual EtO and its by-products (ethylene glycol, ethylene chlorohydrin) in hydrophilic hydrogels may cause cytotoxicity—residual levels must be verified to fall below the allowable limits specified in ISO 10993–7:2008, which specifically addresses EtO residuals in EtO-sterilized devices. Additional sterilization standards apply to gamma irradiation (ISO 11137) and aseptic processing (ISO 13408). Sterile filtration is only applicable to low-viscosity precursor solutions that can pass through a 0.22 μm filter membrane prior to gelation—for systems containing nanoparticles (AgNPs, Pg-Cu(II) ICPs) or high-molecular-weight polymers, filtration may retain functional components (Wang et al., 2025).

Regulatory complexity of combination products (based on Wang et al. (2025)): Multifunctional POPF hydrogels that simultaneously contain a medical device component (physical barrier) and a drug/bioactive factor will be classified by regulatory agencies as “combination products.” Wang et al. (Wang et al., 2025) noted that such products “may require 7–12 years from laboratory research to obtaining regulatory marketing authorization” [as cited in 66], and “in the United States, average costs of hundreds of millions of dollars to obtain market approval.” The specific regulatory pathway—510(k), Premarket Approval (PMA), Biologics License Application (BLA), or a combination product designation—depends on the product’s intended use, composition, primary mode of action, release characteristics, and formal determination by the relevant regulatory agency. It is premature to assign a specific route to multifunctional hydrogel systems solely because they incorporate a bioactive molecule.

Inferred regulatory classifications for each POPF hydrogel system (requiring confirmation with regulatory agencies): Pure physical barrier hydrogels (PVA sheet, Kemmochi et al. (2021)) may be classifiable as medical devices. AgNP-containing antibacterial hydrogels (Zhou et al. (2026))—the antibacterial mechanism of AgNPs and the specific regulatory requirements for nanomaterials may lead to classification complexity. Meropenem-containing hydrogels (Zhou et al. (2025)) clearly involve a drug component, with high likelihood of combination product classification. Immunotherapeutic agent-containing hydrogels (Zhao et al. (Pausch et al., 2022)) incorporate biological products (IL-15 and anti-TIGIT antibody), potentially requiring a combination product framework with biological product components. Penicillin G-Cu(II) nanodrug-containing hydrogels (Shen et al. (2025)) are combination products, with the nanodrug representing a non-conventional dosage form. Ceffe bioactive mixture-containing hydrogels (She et al. (2026))—the definition of Ceffe (a composite of growth factors plus exosomes plus ECM proteins) itself constitutes a regulatory classification challenge.

Implications for translational strategy: Engaging in pre-submission meetings with regulatory agencies (e.g., FDA Office of Combination Products) at the early development stage to clarify the preliminary regulatory classification of the product and the required non-clinical/clinical data package is a critical strategy for avoiding approval delays caused by later-stage classification uncertainty. Wang et al. (2025) also noted that “clarifying product attributes and formulating reasonable clinical trial and approval strategies” constitutes a major challenge for hydrogel clinical translation.

### 4.5 The diagnostic-therapeutic closed loop—a neglected complementary dimension

The SmartPAN reported by Pausch et al. (Pausch et al., 2025; Yamashita et al., 2013) represents a unique and complementary dimension of POPF management—intraoperative leak detection—rather than therapy. However, the potential synergy between SmartPAN and therapeutic hydrogels constitutes a translational opportunity entirely overlooked in the published literature.

Core data of SmartPAN: This hydrogel indicator employs the pH indicator bromothymol blue (BTB) as the active component and a polysaccharide microsphere matrix as the carrier. Through *in vitro* screening of 41 prototypes and *ex vivo* validation using porcine pancreatic tissue and human pancreatic fluid, the BTB-polysaccharide microsphere system was ultimately selected (Yamashita et al., 2013). In a porcine model (n = 20, approximately 30 kg), SmartPAN demonstrated sensitive and specific instantaneous color reactions, enabling intraoperative targeted closure (Yamashita et al., 2013). The first-in-human clinical trial—the ViP trial (IDEAL Stage I)—enrolled 29 patients undergoing partial pancreatectomy: positive intraoperative leak reactions were detected in 10 of 29 cases; all 16 participating surgeons unanimously rated SmartPAN as “easy to learn and use,” with the majority agreeing it was “useful” and expressing willingness to use it frequently; no adverse events or complications were attributable to SmartPAN; POPF occurred in 7 patients (2 with intraoperatively detected leaks, 5 without detected leaks) (Pausch et al., 2025). These clinical data should be interpreted as preliminary safety and usability evidence from a small, single-arm study (IDEAL Stage I). Human diagnostic sensitivity and specificity were not formally established, and performance demonstrated in pigs should not be assumed to transfer directly to humans. Earlier work by Yamashita et al. (Diamond and Freeman, 2001) on chymotrypsin-activated fluorescent probes and by Mori et al. (Mori et al., 2015) on intraoperative visualization of pancreatic juice leakage provided important foundational concepts for intraoperative leak detection.

Potential synergy with therapeutic hydrogels (hypothetical): Integrating SmartPAN intraoperative leak detection with targeted therapeutic hydrogel sealing into a “diagnostic-therapeutic closed loop” could shift POPF management from the current paradigm of “empirical broad coverage” (routine application of sealants to all pancreatic remnants) toward “detection-driven precision sealing” (targeted treatment only at sites with confirmed leakage). Conceptually, this integration resembles “theranostics” in oncology—a single platform combining diagnostic and therapeutic functions. In adjacent fields, the integration of fluorescence imaging with pH-responsive drug release has been demonstrated at the molecular level; for instance, Zn(II)-Schiff base complexes with lysosome-targeted imaging and pH-triggered camptothecin release illustrate how cyano-modulated donor-acceptor topology can enable combined bioimaging and microenvironment-responsive chemotherapy within a single coordination platform (Zhang et al., 2026). While such systems were not designed for POPF or surgical applications, their conceptual framework—co-localizing diagnostic signal with controlled therapeutic release—provides methodological precedent for the diagnostic-therapeutic integration envisioned in a POPF context. However, critical prerequisites for validating this hypothesis in the POPF setting include: whether the color reaction of SmartPAN would affect the adhesion or crosslinking of therapeutic hydrogels; the impact of the time delay between detection and treatment on sealing efficacy; and the avoidance of potential chemical interactions between SmartPAN residue (polysaccharide microspheres) and hydrogel components (Pausch et al., 2025; Yamashita et al., 2013). Moreover, a diagnostic indicator followed by a separately applied sealant should not be characterized as a clinically validated theranostic platform—it represents two sequential procedures rather than an integrated system.

### 4.6 Safety evidence gaps requiring dedicated investigation

Beyond the translational barriers discussed above, several product-specific long-term safety questions remain unaddressed and warrant consolidated discussion. These gaps are particularly relevant for hydrogels that incorporate pharmacologically active or potentially bioaccumulating components:

1. Antimicrobial resistance risk with sustained local antibiotic release: The prolonged, high-concentration local release of meropenem—a carbapenem antibiotic—from PC-OHAD@MP (Zhou et al., 2025) raises concerns regarding antimicrobial stewardship and the potential induction of carbapenem-resistant organisms at the application site. While localized delivery may reduce systemic selective pressure compared to intravenous administration, the sustained sub-inhibitory concentrations that occur during the terminal phase of release could theoretically promote resistance. No current data address this risk.
2. Long-term biodistribution and organ accumulation of AgNPs: PVA/QCS/AgNP (Zhou et al., 2026) releases Ag^+^ ions that exert antibacterial activity, but the fate of AgNPs following hydrogel degradation—including potential translocation to liver, kidney, spleen, and brain, as documented for other nanoparticulate systems—has not been investigated in the context of intraperitoneal pancreatic applications. Silver accumulation in organs can occur with chronic exposure and may be associated with argyria or, at high doses, organ dysfunction.
3. Cytotoxicity window of Cu(II): CGO@Pg-Cu(II) (Shen et al., 2025) employs Cu(II) coordination for both trypsin inhibition and pH-responsive drug delivery. While Cu(II) concentrations were designed not to exceed 350 μg/mL to ensure biocompatibility, the local concentration-time profile at the pancreatic remnant, potential Cu(II) release into systemic circulation, and the narrow therapeutic window between antimicrobial/trypsin-inhibitory activity and cellular toxicity require more detailed characterization.
4. Immunogenicity and metabolic fate of D-peptides: CP-CNDS (He et al., 2024) utilizes D-amino acid peptides that are naturally resistant to proteolytic degradation—an asset for enzyme resistance but a potential liability if D-peptide fragments accumulate in tissues or elicit immune responses over extended periods. The metabolic clearance pathways for D-amino acid polymers, their potential for immunogenic presentation, and their long-term tissue residence have not been characterized in the pancreatic context.
5. Ceffe batch consistency and biological variability: Ceffe (She et al., 2026) is a complex biological mixture whose composition may vary depending on donor characteristics, adipose tissue source, processing protocols, and storage conditions. This variability may affect both the reproducibility of its therapeutic effects and the ability to standardize its quality for regulatory approval. Defining critical quality attributes for such biologically derived components remains an unresolved challenge.

These safety gaps do not diminish the promise of multifunctional hydrogel strategies; rather, they define the preclinical data package that will be required for each system before regulatory approval for first-in-human trials can be contemplated.

## 5 Conclusions and perspectives

### 5.1 Core conclusions: overall assessment of evidence level

The evidence accumulated in the published literature delineates an emerging trajectory: from the pure PVA physical barrier of Kemmochi et al. (2021) (evidence level: proof-of-concept in a single rat model), to the active enzyme-capturing strategy of He et al. (2024) (elevating sealing from “defense” to “offense”), and onward to the full-cycle temporal management of Shen et al. (2025) and the asymmetric functional zoning of She et al. (2026)—the field has advanced toward “active microenvironment remodeling.” However, all reviewed *in vivo* studies represent preclinical proof-of-concept investigations conducted in small animal models, predominantly in rats. The design limitations inherent to these studies—including unreported sample sizes, short observation periods, heterogeneous endpoints, and the absence of large-animal validation—preclude direct clinical inference. SmartPAN (Pausch et al., 2025) is the only POPF-related hydrogel device to have entered human trials (IDEAL Stage I), but its function is diagnostic leak detection rather than therapeutic sealing. Its clinical evidence constitutes preliminary safety and usability data from a single-arm study, and human diagnostic accuracy has not been formally established.

### 5.2 Future directions

Based on the foregoing analysis, the following directions, ranked by priority, warrant the most urgent research investment.

First priority—large-animal validation and standardized assessment systems. Establish a POPF hydrogel evaluation platform in a porcine model, which should at minimum include: (1) a standardized PF induction method; (2) unified endpoint metrics (time course of drain amylase/lipase, burst pressure, histological healing scores, inflammatory cytokine temporal profiles); and (3) a minimum of 30-day follow-up to assess long-term safety and degradation behavior. The establishment of this platform is a prerequisite for any multifunctional hydrogel system to enter human trials. Multiple publications have called for large-animal validation (Shen et al., 2025; Zhou et al., 2025; Pausch et al., 2022), yet none has been executed to date for multifunctional therapeutic hydrogels. Methodologically, future animal studies should explicitly report sample sizes and the basis for sample size estimation, to enhance the reproducibility of studies and the evaluability of evidence.

Second priority—establishment of a degradation-healing simultaneous monitoring model. Within the same porcine model, employ methods such as non-invasive fluorescence imaging (Art et al., 2011) to simultaneously track the temporal changes of hydrogel degradation, anastomotic mechanical strength, and histological healing, thereby establishing quantitative criteria for “degradation-healing matching” based on experimental data rather than theoretical extrapolation. Such studies must incorporate functional measures of sealing integrity (adhesion retention, burst resistance) alongside mass retention data.

Third priority—evaluation of minimally invasive surgical adaptability. Using laparoscopic simulators and porcine minimally invasive models (Kendrick and Cusati, 2010; Giulianotti et al., 2010), systematically evaluate the delivery feasibility, positioning accuracy, and crosslinking efficiency of existing hydrogel systems (injectable vs. pre-formed patch vs. sprayable) in the laparoscopic environment, identifying and resolving specific technical barriers to procedural adaptability. Particular attention should be paid to UVA irradiation uniformity in the laparoscopic setting and the operative time window constraints imposed by gelation kinetics.

Hypothetical direction—computational assistance for materials design (to be validated). He et al. (2024) have demonstrated the feasibility of molecular dynamics simulations for screening D-peptide enzyme-capturing motifs. Wang et al. (2025) proposed that artificial intelligence and big data analytics could “design more complex hydrogels.” The application of these computational tools to the optimization of anti-enzymatic peptide sequences, performance prediction of multifunctional hydrogel compositions, and rational design of degradation profiles represents a direction worth exploring. However, at present, this direction has no experimental validation whatsoever within the POPF hydrogel domain and constitutes only a methodological extrapolation based on advances in adjacent fields.

Hypothetical direction—diagnostic-therapeutic integration (to be validated). SmartPAN (Pausch et al., 2025; Yamashita et al., 2013) has provided preliminary clinical safety and usability data supporting intraoperative pancreatic leak detection in an IDEAL Stage I setting (n = 29). Should the integration of SmartPAN intraoperative leak detection with targeted therapeutic hydrogel sealing be validated as feasible by subsequent preclinical studies—i.e., demonstrating chemical and functional compatibility of the two hydrogel components and that the detection-to-treatment time window does not compromise sealing efficacy—it could potentially advance POPF management from empirical broad coverage toward precision targeted sealing. However, it must be emphasized that no integrated diagnostic-therapeutic study has been published to date, and this direction remains at the hypothesis stage.

## References

[B1] Adelmeijer J. Porte R. J. Lisman T. (2013). *In vitro* effects of proteases in human pancreatic juice on stability of liquid and carrier-bound fibrin sealants. Br. J. Surg. 100 (11), 1498–1504. 10.1002/bjs.9243 24037572

[B2] Akita H. Takahashi H. Gotoh K. Kobayashi S. Sugimura K. Miyoshi N. (2015). Closure method for thick pancreas stump after distal pancreatectomy: soft coagulation and polyglycolic acid felt with fibrin glue. Langenbecks Arch. Surg. 400, 843–848. 10.1007/s00423-015-1339-2 26345639

[B3] Allen P. J. Gönen M. Brennan M. F. Bucknor A. A. Robinson L. M. Pappas M. M. (2014). Pasireotide for postoperative pancreatic fistula. N. Engl. J. Med. 370 (21), 2014–2022. 10.1056/NEJMoa1313688 24849084

[B4] Artzi N. Oliva N. Puron C. Shitreet S. Artzi S. bon Ramos A. (2011). *In* vivo and in vitro tracking of erosion in biodegradable materials using non-invasive fluorescence imaging. Nat. Mater 10 (9), 704–709. 10.1038/nmat3095 21857678 PMC3160718

[B5] Balcom J. H. 4th Rattner D. W. Warshaw A. L. Chang Y. Fernandez-del Castillo C. (2001). Ten-year experience with 733 pancreatic resections: changing indications, older patients, and decreasing length of hospitalization. Arch. Surg. 136 (4), 391–398. 10.1001/archsurg.136.4.391 11296108

[B6] Balcom J. H. 4th Keck T. Warshaw A. L. Graeme-Cook F. Fernández-del Castillo C. (2002). Prevention of pancreatic fistula with a new synthetic, absorbable sealant: evaluation in a dog model. J. Am. Coll. Surg. 195 (4), 490–496. 10.1016/S1072-7515(02)01313-3 12375754

[B7] Bassi C. Dervenis C. Butturini G. Fingerhut A. Yeo C. Izbicki J. (2005). Postoperative pancreatic fistula: an international study group (ISGPF) definition. Surgery 138 (1), 8–13. 10.1016/j.surg.2005.05.001 16003309

[B8] Bassi C. Marchegiani G. Dervenis C. Sarr M. Abu Hilal M. Adham M. (2017). The 2016 update of the International Study Group (ISGPS) definition and grading of postoperative pancreatic fistula: 11 years after. Surgery 161 (3), 584–591. 10.1016/j.surg.2016.11.014 28040257

[B9] Büchler M. W. Friess H. Klempa I. Hermanek P. Sulkowski U. Becker H. (1992). Role of octreotide in the prevention of postoperative complications following pancreatic resection. Am. J. Surg. 163 (1), 125–131. 10.1016/0002-9610(92)90264-r 1733360

[B10] Büchler M. W. Wagner M. Schmied B. M. Uhl W. Friess H. Z'graggen K. (2003). Changes in morbidity after pancreatic resection: toward the end of completion pancreatectomy. Arch. Surg. 138 (12), 1310–1314. 10.1001/archsurg.138.12.1310 14662530

[B11] Callery M. P. Pratt W. B. Kent T. S. Chaikof E. L. Vollmer C. M. Jr (2013). A prospectively validated clinical risk score accurately predicts pancreatic fistula after pancreatoduodenectomy. J. Am. Coll. Surg. 216 (1), 1–14. 10.1016/j.jamcollsurg.2012.09.002 23122535

[B12] Cameron J. L. He J. (2015). Two thousand consecutive pancreaticoduodenectomies. J. Am. Coll. Surg. 220 (4), 530–536. 10.1016/j.jamcollsurg.2014.12.031 25724606

[B13] Carter T. I. Fong Z. V. Hyslop T. Lavu H. Tan W. P. Hardacre J. (2013). A dual-institution randomized controlled trial of remnant closure after distal pancreatectomy: does the addition of a falciform patch and fibrin glue improve outcomes? J. Gastrointest. Surg. 17, 102–109. 10.1007/s11605-012-1963-x 22798186

[B14] Cheng Y. Briarava M. Lai M. Wang X. Tu B. Cheng N. (2017). Pancreaticojejunostomy versus pancreaticogastrostomy reconstruction for the prevention of postoperative pancreatic fistula following pancreaticoduodenectomy. Cochrane Database Syst. Rev. 9 (9), CD012257. 10.1002/14651858.CD012257.pub2 28898386 PMC6483797

[B15] Conaglen P. J. Collier N. A. (2014). Augmenting pancreatic anastomosis during Whipple operation with fibrin glue: a beneficial technical modification? ANZ J. Surg. 84 (7-8), 558–562. 10.1111/ans.12072 23418951

[B16] D'Andrea A. A. Costantino V. Sperti C. Pedrazzoli S. (1994). Human fibrin sealant in pancreatic surgery: it is useful in preventing fistulas? A prospective randomized study. Ital. J. Gastroenterol. 26, 283–286. 7949264

[B17] DeOliveira M. L. Winter J. M. Schafer M. Cunningham S. C. Cameron J. L. Yeo C. J. (2006). Assessment of complications after pancreatic surgery: a novel grading system applied to 633 patients undergoing pancreaticoduodenectomy. Ann. Surg. 244 (6), 931–937. 10.1097/01.sla.0000246856.03918.9a 17122618 PMC1856636

[B18] Diamond M. P. Freeman M. L. (2001). Clinical implications of postsurgical adhesions. Hum. Reprod. Update 7 (6), 567–576. 10.1093/humupd/7.6.567 11727865

[B19] Diener M. K. Seiler C. M. Rossion I. Kleeff J. Glanemann M. Butturini G. (2011). Efficacy of stapler versus hand-sewn closure after distal pancreatectomy (DISPACT): a randomised, controlled multicentre trial. Lancet 377 (9776), 1514–1522. 10.1016/S0140-6736(11)60237-7 21529927

[B20] Dong Z. Xu J. Wang Z. Petrov M. S. (2016). Stents for the prevention of pancreatic fistula following pancreaticoduodenectomy. Cochrane Database Syst. Rev. (5), CD008914. 10.1002/14651858.CD008914.pub2 27153248 PMC7156907

[B21] Ecker B. L. McMillan M. T. Allegrini V. Bassi C. Beane J. D. Beckman R. M. (2019). Risk factors and mitigation strategies for pancreatic fistula after distal pancreatectomy: analysis of 2026 resections from the international, multi-institutional distal pancreatectomy study group. Ann. Surg. 269 (1), 143–149. 10.1097/SLA.0000000000002491 28857813

[B22] Elayat A. A. el-Naggar M. M. Tahir M. (1995). An immunocytochemical and morphometric study of the rat pancreatic islets. J. Anat. 186, 629–637. 7559135 PMC1167020

[B23] Ellis-Behnke R. G. Liang Y. X. Tay D. K. Kau P. W. F. Schneider G. E. Zhang S. (2006). Nano hemostat solution: immediate hemostasis at the nanoscale. Nanomedicine 2 (4), 207–215. 10.1016/j.nano.2006.08.001 17292144

[B24] Ferrone C. R. Warshaw A. L. Rattner D. W. Berger D. Zheng H. Rawal B. (2008). Pancreatic fistula rates after 462 distal pancreatectomies: staplers do not decrease fistula rates. J. Gastrointest. Surg. 12 (10), 1691–1697. 10.1007/s11605-008-0636-2 18704597 PMC3806097

[B25] Fujii T. Sugimoto H. Yamada S. Kanda M. Suenaga M. Takami H. (2014). Modified Blumgart anastomosis for pancreaticojejunostomy: technical improvement in matched historical control study. J. Gastrointest. Surg. 18 (6), 1108–1115. 10.1007/s11605-014-2523-3 24733259

[B26] Giulianotti P. C. Sbrana F. Bianco F. M. Elli E. F. Shah G. Addeo P. (2010). Robot-assisted laparoscopic pancreatic surgery: single-surgeon experience. Surg. Endosc. 24 (7), 1646–1657. 10.1007/s00464-009-0825-4 20063016

[B27] Goetz M. R. Heumann A. Bassi C. Landoni L. Khatib-Chahidi K. Ghadimi M. (2024). Safety and performance of a synthetic sealant patch aimed to prevent postoperative pancreatic fistula after distal pancreatectomy (SHIELDS) - Prospective international multicenter phase II study. HPB (Oxford) 26 (7), 903–910. 10.1016/j.hpb.2024.03.002 38653711

[B28] Gong J. He S. Cheng Y. Cheng N. Gong J. Zeng Z. (2018). Fibrin sealants for the prevention of postoperative pancreatic fistula following pancreatic surgery. Cochrane Database Syst. Rev. (6), CD009621. 10.1002/14651858.cd009621.pub3 29934987 PMC6513198

[B29] Gurusamy K. S. Koti R. Fusai G. Davidson B. R. (2013). Somatostatin analogues for pancreatic surgery. Cochrane Database Syst. Rev. (4), CD008370. 10.1002/14651858.CD008370.pub2 23633353 PMC7175835

[B30] Hackert T. Hinz U. Pausch T. Fesenbeck I. Strobel O. Schneider L. (2016). Postoperative pancreatic fistula: we need to redefine grades B and C. Surgery 159, 877–882. 10.1016/j.surg.2015.09.014 26603847

[B31] Hartwig W. Hackert T. Hinz U. Gluth A. Bergmann F. Strobel O. (2011). Pancreatic cancer surgery in the new millennium: better prediction of outcome. Ann. Surg. 254, 311–319. 10.1097/SLA.0b013e31821fd334 21606835

[B32] He W. Wang Y. Li X. Ji Y. Yuan J. Yang W. (2024). Sealing the Pandora’s vase of pancreatic fistula through entrapping the digestive enzymes within a dextrorotary (D)-peptide hydrogel. Nat. Commun. 15 (1), 7235. 10.1038/s41467-024-51734-7 39174548 PMC11341566

[B33] Hirono S. Kawai M. Okada K. I. Miyazawa M. Kitahata Y. Hayami S. (2019). Modified Blumgart mattress suture versus conventional interrupted suture in pancreaticojejunostomy during pancreaticoduodenectomy: randomized controlled trial. Ann. Surg. 269 (2), 243–251. 10.1097/SLA.0000000000002802 29697455 PMC6325750

[B34] Hüttner F. J. Mihaljevic A. L. Hackert T. Ulrich A. Büchler M. W. Diener M. K. (2016). Effectiveness of Tachosil in the prevention of pancreatic fistula after distal pancreatectomy: a systematic review and meta-analysis. Langenbecks Arch. Surg. 401 (2), 151–159. 10.1007/s00423-016-1382-7 26897031

[B35] Kawaida H. Kono H. Watanabe M. Hosomura N. Amemiya H. Fujii H. (2018). Risk factors of postoperative pancreatic fistula after distal pancreatectomy using a triple-row stapler. Surg. Today 48 (1), 95–100. 10.1007/s00595-017-1554-2 28600634 PMC5711995

[B36] Kawaida H. Kono H. Hosomura N. Amemiya H. Itakura J. Fujii H. (2019). Surgical techniques and postoperative management to prevent postoperative pancreatic fistula after pancreatic surgery. World J. Gastroenterol. 25, 3722–3737. 10.3748/wjg.v25.i28.3722 31391768 PMC6676555

[B50] Kawai M. Hirono S. Okada K. I. Satoi S. Yanagimoto H. Kon M. (2017). Reinforced staplers for distal pancreatectomy. Langenbecks Arch. Surg. 402 (8), 1197–1204. 10.1007/s00423-017-1634-1 29103084

[B37] Kemmochi A. Tamura T. Shimizu Y. Owada Y. Ozawa Y. Hisakura K. (2021). A novel hydrogel sheet prevents postoperative pancreatic fistula in a rat model. J. Hepatobiliary Pancreat. Sci. 28 (2), 192–201. 10.1002/jhbp.867 33179402 PMC7984228

[B38] Kendrick M. L. Cusati D. (2010). Total laparoscopic pancreaticoduodenectomy: feasibility and outcome in an early experience. Arch. Surg. 145 (1), 19–23. 10.1001/archsurg.2009.243 20083750

[B39] Kleeff J. Diener M. K. Z'graggen K. Hinz U. Wagner M. Bachmann J. (2007). Distal pancreatectomy: risk factors for surgical failure in 302 consecutive cases. Ann. Surg. 245 (4), 573–582. 10.1097/01.sla.0000251438.43135.fb PMC1877036 17414606

[B40] Li J. Celiz A. D. Yang J. Yang Q. Wamala I. Whyte W. (2017). Tough adhesives for diverse wet surfaces. Science 357 (6349), 378–381. 10.1126/science.aah6362 28751604 PMC5905340

[B41] Lillemoe K. D. Cameron J. L. Kim M. P. Campbell K. A. Sauter P. K. Coleman J. A. (2004). Does fibrin glue sealant decrease the rate of pancreatic fistula after pancreaticoduodenectomy? Results of a prospective randomized trial. J. Gastrointest. Surg. 8, 766–772. 10.1016/j.gassur.2004.06.011 15531229

[B42] Malka D. Hammel P. Sauvanet A. Rufat P. O'Toole D. Bardet P. (2000). Risk factors for diabetes mellitus in chronic pancreatitis. Gastroenterology 119 (5), 1324–1332. 10.1053/gast.2000.19286 11054391

[B43] Martin I. Au K. (2013). Does fibrin glue sealant decrease the rate of anastomotic leak after a pancreaticoduodenectomy? Results of a prospective randomized trial. HPB Oxf. 15, 561–566. 10.1111/hpb.12018 23458447 PMC3731575

[B44] McMillan M. T. Christein J. D. Callery M. P. Behrman S. W. Drebin J. A. Hollis R. H. (2016). Comparing the burden of pancreatic fistulas after pancreatoduodenectomy and distal pancreatectomy. Surgery 159 (4), 1013–1022. 10.1016/j.surg.2015.10.028 26670325

[B45] Mikamori M. Gotoh K. Kobayashi S. Uesugi K. Iwagami Y. Yamada D. (2022). Self-assembling peptide hydrogel SPG-178 as a pancreatic fistula-preventing agent. Langenbecks Arch. Surg. 407 (1), 189–196. 10.1007/s00423-021-02226-5 34100123

[B46] Miller B. C. Christein J. D. Behrman S. W. Drebin J. A. Pratt W. B. Callery M. P. (2014). A multi-institutional external validation of the fistula risk score for pancreatoduodenectomy. J. Gastrointest. Surg. 18 (1), 172–180. 10.1007/s11605-013-2337-8 24002771

[B47] Montorsi M. Zerbi A. Bassi C. Capussotti L. Coppola R. Sacchi M. (2012). Efficacy of an absorbable fibrin sealant patch (TachoSil) after distal pancreatectomy: a multicenter, randomized, controlled trial. Ann. Surg. 256, 853–860. 10.1097/SLA.0b013e318272dec0 23095631

[B48] Mori K. Ishizawa T. Yamashita S. Kamiya M. Urano Y. Kokudo N. (2015). Intraoperative visualization of pancreatic juice leaking from the pancreatic stump in a swine model. Gastroenterology 149, 1334–1336. 10.1053/j.gastro.2015.07.068 26363217

[B49] Motoi F. Egawa S. Rikiyama T. Katayose Y. Unno M. (2012). Randomized clinical trial of external stent drainage of the pancreatic duct to reduce postoperative pancreatic fistula after pancreaticojejunostomy. Br. J. Surg. 99 (4), 524–531. 10.1002/bjs.8654 22497024

[B51] Nahm C. B. Connor S. J. Samra J. S. Mittal A. (2018). Postoperative pancreatic fistula: a review of traditional and emerging concepts. Clin. Exp. Gastroenterol. 11, 105–118. 10.2147/CEG.S120217 29588609 PMC5858541

[B52] Nanashima A. Tobinaga S. Kunizaki M. Nonaka T. Takeshita H. Hidaka S. (2012). Does fibrin glue prevent biliary and pancreatic fistula after surgical resection? Hepatogastroenterology 59 (117), 1544–1547. 10.5754/hge10079 22683970

[B53] Nathan H. Cameron J. L. Goodwin C. R. Seth A. K. Edil B. H. Wolfgang C. L. (2009). Risk factors for pancreatic leak after distal pancreatectomy. Ann. Surg. 250 (2), 277–281. 10.1097/SLA.0b013e3181ae34be 19638926

[B54] Orci L. A. Oldani G. Berney T. Andres A. Mentha G. Morel P. (2014). Systematic review and meta-analysis of fibrin sealants for patients undergoing pancreatic resection. HPB Oxf. 16 (1), 3–11. 10.1111/hpb.12064 23461684 PMC3892308

[B55] Pausch T. M. Bartel M. Cui J. Aubert O. Mitzscherling C. Liu X. (2022). SmartPAN: in vitro and in vivo proof-of-safety assessments for an intra-operative predictive indicator of postoperative pancreatic fistula. Basic Clin. Pharmacol. Toxicol. 130 (4), 542–552. 10.1111/bcpt.13708 35040273

[B56] Pausch T. M. Holze M. El-Mahdy J. Gesslein B. Thedius H. O. Sander A. (2025). Visualization of intraoperative pancreatic leakage (ViP): the IDEAL stage I first-in-human, single-arm clinical pilot trial of SmartPAN. Ann. Surg. Open 6 (1), e529. 10.1097/AS9.0000000000000529 40134475 PMC11932625

[B57] Pavlik M. I. Røsok B. I. Kazaryan A. M. Rosseland A. R. Edwin B. (2011). Effect of TachoSil patch in prevention of postoperative pancreatic fistula. J. Gastrointest. Surg. 15, 1625–1629. 10.1007/s11605-011-1584-9 PMC3159765 21671113

[B58] Peng S. Y. Wang J. W. Lau W. Y. Cai X. J. Mou Y. P. Liu Y. B. (2007). Conventional versus binding pancreaticojejunostomy after pancreaticoduodenectomy: a prospective randomized trial. Ann. Surg. 245 (5), 692–698. 10.1097/01.sla.0000255588.50964.5d 17457161 PMC1877076

[B59] Pessaux P. Sauvanet A. Mariette C. Paye F. Muscari F. Cunha A. S. (2011). External pancreatic duct stent decreases pancreatic fistula rate after pancreaticoduodenectomy: prospective multicenter randomized trial. Ann. Surg. 253 (5), 879–885. 10.1097/sla.0b013e31821219af 21368658

[B60] Privett B. J. Perini M. V. Weinberg L. Fink M. A. Muralidharan V. Lee E. (2021). Reduction in post-operative pancreatic fistula with polyethylene glycol and recombinant human albumin sealant following stapled distal pancreatectomy. ANZ J. Surg. 91 (11), 2459–2465. 10.1111/ans.17181 34514684

[B61] Roberts K. J. Sutcliffe R. P. Marudanayagam R. Hodson J. Isaac J. Muiesan P. (2015). Scoring System to predict pancreatic fistula after pancreaticoduodenectomy: a UK multicenter study. Ann. Surg. 261 (6), 1191–1197. 10.1097/SLA.0000000000000997 25371115

[B62] Satoi S. Toyokawa H. Yanagimoto H. Yamamoto T. Hirooka S. Yui R. (2011). Reinforcement of pancreaticojejunostomy using polyglycolic acid mesh and fibrin glue sealant. Pancreas 40, 16–20. 10.1097/MPA.0b013e3181f82f55 20966808

[B63] Serradilla-Martín M. Paterna-López S. Palomares-Cano A. Cantalejo-Díaz M. Abadía-Forcén T. Gutiérrez-Díez M. L. (2023). Polyethylene glycol-coated haemostatic patch for prevention of clinically relevant postoperative pancreatic fistula after pancreatoduodenectomy: randomized clinical trial. BJS Open 7 (2), zrad028. 10.1093/bjsopen/zrad028 PMC10077024 37021546

[B64] She H. Shao H. Chen W. Zhou Y. Ma J. Xu Z. (2026). A multifunctional bilayer hydrogel for effective pancreatic fistula prevention and post-surgical adhesion control. Adv. Health Mater. 15 (11), e03972. 10.1002/adhm.202503972 41388685

[B65] Shen S. He Y. Yan W. Zeng D. Liu L. Dong Z. (2025). Light-activated transient unilateral adhesive recombinant collagen-based hydrogel for reversible fibrosis induction and full-cycle management of pancreatic fistula. Adv. Mater. 37 (39), e2506907. 10.1002/adma.202506907 40643059

[B66] Shrikhande S. V. Sivasanker M. Vollmer C. M. Friess H. Besselink M. G. Fingerhut A. (2017). Pancreatic anastomosis after pancreatoduodenectomy: a position statement by the International Study Group of pancreatic Surgery (ISGPS). Surgery 161 (5), 1221–1234. 10.1016/j.surg.2016.11.021 28027816

[B67] Siegel R. L. Miller K. D. Jemal A. (2017). Cancer Statistics, 2017. CA Cancer J. Clin. 67, 7–30. 10.3322/caac.21387 28055103

[B68] Suc B. Msika S. Fingerhut A. Fourtanier G. Hay J. M. Holmières F. (2003). Temporary fibrin glue occlusion of the main pancreatic duct in the prevention of intra-abdominal complications after pancreatic resection: prospective randomized trial. Ann. Surg. 237, 57–65. 10.1097/00000658-200301000-00009 12496531 PMC1513966

[B69] Sugimoto M. Gotohda N. Kato Y. Takahashi S. Kinoshita T. Shibasaki H. (2013). Risk factor analysis and prevention of postoperative pancreatic fistula after distal pancreatectomy with stapler use. J. Hepatobiliary Pancreat. Sci. 20 (5), 538–544. 10.1007/s00534-013-0596-0 23430057

[B70] Sun W. Zhang X. Zhao Y. Ye B. Gao W. Jiang G. (2026). pH/NIR dual-responsive implantable patch for combined photothermal and chemotherapy of tumors. Int. J. Pharm. X 11, 100582. 10.1016/j.ijpx.2026.100582 PMC13262176 42292306

[B71] Suzuki Y. Kuroda Y. Morita A. Fujino Y. Tanioka Y. Kawamura T. (1995). Fibrin glue sealing for the prevention of pancreatic fistulas following distal pancreatectomy. Arch. Surg. 130 (9), 952–955. 10.1001/archsurg.1995.01430090038015 7661678

[B72] Tanaka T. Kuroki T. Adachi T. Ono S. Kitasato A. Hirabaru M. (2013). Development of a novel rat model with pancreatic fistula and the prevention of this complication using tissue-engineered myoblast sheets. J. Gastroenterol. 48 (9), 1081–1089. 10.1007/s00535-012-0706-9 23179607

[B73] Vollmer C. M. Jr Sanchez N. Gondek S. McAuliffe J. Kent T. S. Christein J. D. (2012). A root-cause analysis of mortality following major pancreatectomy. J. Gastrointest. Surg. 16 (1), 89–102. 10.1007/s11605-011-1753-x 22065319

[B74] Wang Y. Liu Z. Zhang W. Wang Y. Gao S. Li X. (2025). Hydrogel-based delivery systems for pancreatic cancer therapy: from bench to bedside. Mater Today Bio 29, 101328. 10.1016/j.mtbio.2025.101328

[B75] Winter J. M. Cameron J. L. Campbell K. A. Arnold M. A. Chang D. C. Coleman J. (2006). 1423 pancreaticoduodenectomies for pancreatic cancer: a single-institution experience. J. Gastrointest. Surg. 10 (9), 1199–1210. 10.1016/j.gassur.2006.08.018 17114007

[B76] Wu W. He J. Cameron J. L. Makary M. Soares K. Ahuja N. (2014). The impact of postoperative complications on the administration of adjuvant therapy following pancreaticoduodenectomy for adenocarcinoma. Ann. Surg. Oncol. 21 (9), 2873–2881. 10.1245/s10434-014-3722-6 24770680 PMC4454347

[B77] Xu L. Bai E. Zhu Y. Qin J. Du X. Huang H. (2023a). pH-Responsive hydrogel as a potential oral delivery system of baicalin for prolonging gastroprotective activity. Pharmaceutics 15, 257. 10.3390/pharmaceutics15010257 36678886 PMC9861087

[B78] Xu L. Du X. Zhou Y. Cao X. Shen Y. Zhu H. (2023b). Polyaspartic acid-stabilized CaCO_3_-containing in situ hydrogel for protection and treatment of gastric ulcer. Mol. Pharm. 20 (4), 2105–2118. 10.1021/acs.molpharmaceut.2c01062 36916647

[B79] Yamashita S. Sakabe M. Ishizawa T. Hasegawa K. Urano Y. Kokudo N. (2013). Visualization of the leakage of pancreatic juice using a chymotrypsin-activated fluorescent probe. Br. J. Surg. 100, 1220–1228. 10.1002/bjs.9185 23765524

[B80] Yeo C. J. Cameron J. L. Lillemoe K. D. Sauter P. K. Coleman J. Sohn T. A. (2000). Does prophylactic octreotide decrease the rates of pancreatic fistula and other complications after pancreaticoduodenectomy? Results of a prospective randomized placebo-controlled trial. Ann. Surg. 232 (3), 419–429. 10.1097/00000658-200009000-00014 10973392 PMC1421155

[B81] Yuk H. Wu J. Zhao X. (2022). Hydrogel interfaces for merging humans and machines. Nat. Rev. Mater 7, 935–952. 10.1038/s41578-022-00483-4

[B82] Zhang H. Zhu F. Shen M. Tian R. Shi C. J. Wang X. (2015). Systematic review and meta-analysis comparing three techniques for pancreatic remnant closure following distal pancreatectomy. Br. J. Surg. 102 (1), 4–15. 10.1002/bjs.9653 25388952

[B83] Zhang H. Zhou P. Jiang Y. Li L. Ju F. Cheng Q. (2023). Sustained-release esketamine based nanoparticle-hydrogel delivery system for neuropathic pain management. Int. J. Nanomedicine 18, 1131–1143. 10.2147/IJN.S400798 PMC10007983 36915698

[B84] Zhang X. Sun W. Li X. Li Y. Xiang S. Zhang L. (2026). Cyano-modulated Zn(II)-Schiff base complexes for lysosome-targeted fluorescence imaging and pH-responsive camptothecin delivery. Int. J. Pharm. X 12, 100594. 10.1016/j.ijpx.2026.100594 42434439 PMC13351152

[B85] Zhang K. Chen X. Xue Y. Lin J. Liang X. Zhang J. (2022). Tough hydrogel bioadhesives for sutureless wound sealing, hemostasis and biointerfaces. Adv. Funct. Mater. 32, 2111465. 10.1002/adfm.202111465

[B86] Zhao R. Xiao Q. Wu Y. Zhang W. Liu J. Zeng Y. (2024). Dual-crosslinking immunostimulatory hydrogel synchronously suppresses pancreatic fistula and pancreatic cancer relapse post-resection. Biomaterials 305, 122453. 10.1016/j.biomaterials.2023.122453 38159361

[B87] Zhou W. Lv R. Qi W. Mou Y. Cai X. Herr I. (2010). Stapler vs suture closure of pancreatic remnant after distal pancreatectomy: a meta-analysis. Am. J. Surg. 200 (4), 529–536. 10.1016/j.amjsurg.2009.12.022 20538249

[B88] Zhou Y. Li L. Chen F. Huang T. Pan M. Huang H. (2025). Multiaction antimicrobial, anti-inflammatory, and prohealing hydrogel as a novel strategy for preventing postoperative pancreatic fistula. Biomater. Res. 29, 0194. 10.34133/bmr.0194 40271421 PMC12015097

[B89] Zhou Y. Chen F. Lin R. Huang H. Pan M. (2026). A novel PVA/QCS/AgNP hydrogel with antibacterial and healing-promoting efficacy for preventing postoperative pancreatic fistula in rats. Colloids Surf. B Biointerfaces 258, 115251. 10.1016/j.colsurfb.2025.115251 41207062

